# Extracellular vesicles from immortalized mesenchymal stromal cells protect against neonatal hypoxic-ischemic brain injury

**DOI:** 10.1186/s41232-023-00274-6

**Published:** 2023-04-17

**Authors:** Nicole Labusek, Yanis Mouloud, Christian Köster, Eva Diesterbeck, Tobias Tertel, Constanze Wiek, Helmut Hanenberg, Peter A. Horn, Ursula Felderhoff-Müser, Ivo Bendix, Bernd Giebel, Josephine Herz

**Affiliations:** 1 Department of Pediatrics I, Neonatology & Experimental Perinatal Neurosciences, Centre for Translational and Behavioral Sciences (C-TNBS), University Hospital Essen, University Duisburg-Essen, Essen, Germany; 2 Institute for Transfusion Medicine, University Hospital Essen, University Duisburg-Essen, Essen, Germany; 3 Department of Otorhinolaryngology and Head/Neck Surgery, University Hospital Düsseldorf, Heinrich-Heine-University Düsseldorf, Düsseldorf, Germany; 4 Department of Pediatrics III, University Hospital Essen, University Duisburg-Essen, Essen, Germany

**Keywords:** Neonatal encephalopathy, Hypoxia–ischemia, Neuroinflammation, Neuroregeneration, Mesenchymal stromal cells, Extracellular vesicles, Exosomes

## Abstract

**Background:**

Human mesenchymal stromal cell (MSC)-derived extracellular vesicles (EV) revealed neuroprotective potentials in various brain injury models, including neonatal encephalopathy caused by hypoxia–ischemia (HI). However, for clinical translation of an MSC-EV therapy, scaled manufacturing strategies are required, which is challenging with primary MSCs due to inter- and intra-donor heterogeneities. Therefore, we established a clonally expanded and immortalized human MSC line (ciMSC) and compared the neuroprotective potential of their EVs with EVs from primary MSCs in a murine model of HI-induced brain injury. In vivo activities of ciMSC-EVs were comprehensively characterized according to their proposed multimodal mechanisms of action.

**Methods:**

Nine-day-old C57BL/6 mice were exposed to HI followed by repetitive intranasal delivery of primary MSC-EVs or ciMSC-EVs 1, 3, and 5 days after HI. Sham-operated animals served as healthy controls. To compare neuroprotective effects of both EV preparations, total and regional brain atrophy was assessed by cresyl-violet-staining 7 days after HI. Immunohistochemistry, western blot, and real-time PCR were performed to investigate neuroinflammatory and regenerative processes. The amount of peripheral inflammatory mediators was evaluated by multiplex analyses in serum samples.

**Results:**

Intranasal delivery of ciMSC-EVs and primary MSC-EVs comparably protected neonatal mice from HI-induced brain tissue atrophy. Mechanistically, ciMSC-EV application reduced microglia activation and astrogliosis, endothelial activation, and leukocyte infiltration. These effects were associated with a downregulation of the pro-inflammatory cytokine IL-1 beta and an elevated expression of the anti-inflammatory cytokines IL-4 and TGF-beta in the brain, while concentrations of cytokines in the peripheral blood were not affected. ciMSC-EV-mediated anti-inflammatory effects in the brain were accompanied by an increased neural progenitor and endothelial cell proliferation, oligodendrocyte maturation, and neurotrophic growth factor expression.

**Conclusion:**

Our data demonstrate that ciMSC-EVs conserve neuroprotective effects of primary MSC-EVs via inhibition of neuroinflammation and promotion of neuroregeneration. Since ciMSCs can overcome challenges associated with MSC heterogeneity, they appear as an ideal cell source for the scaled manufacturing of EV-based therapeutics to treat neonatal and possibly also adult brain injury.

**Supplementary Information:**

The online version contains supplementary material available at 10.1186/s41232-023-00274-6.

## Background

Mesenchymal stromal cells (MSCs) are multipotent progenitor cells with immunomodulatory and regenerative properties whose therapeutic potential has been investigated in more than 1500 registered clinical trials (clinicaltrials.gov). Initially shown in models of acute kidney injury and myocardial infarction [1, 2], MSCs apparently exert most of their therapeutic effects via extracellular vesicles (EVs), including exosomes and microvesicles [3–5]. Importantly, we have previously proven therapeutic effects of MSC-EVs in a patient with treatment-refractory acute graft-versus-host disease (aGvHD) [6]. Furthermore, according to well-documented neuroprotective effects of MSCs in ischemic brain injury models [7–11], we have shown that MSC-EV products provide similar neuroprotection as their parental cells in an adult ischemic stroke model [8]. In addition, the therapeutic potential of MSC-EVs has been demonstrated in several models of perinatal brain injury, including inflammation-induced preterm brain injury and neonatal encephalopathy caused by hypoxia–ischemia (HI) [12–17]. In view of current limitations of the only available therapy for the treatment of HI-induced brain injury, i.e., hypothermia [18, 19], MSC-EV treatment represents a novel therapy to protect the immature brain.

A major challenge for translation of MSC-EV products into clinical practice is the limited expandability, and heterogeneity of primary MSCs. MSC heterogeneities are most likely the cause for discrepant results observed in a number of clinical trials investigating therapeutic effects of MSC therapies especially in aGvHD patients [20–24]. Challenges associated with the cells’ heterogeneity also apply for their MSC-EV products. Our recent preclinical studies demonstrated that due to inter- and intra-donor MSC heterogeneity, only a proportion of MSC-EV products provide therapeutic effects [25–27]. Even though manufactured with standard procedures from the same MSC stocks, we observed pronounced batch-to-batch variations of EV products [27–29]. MSC heterogeneity paired with the limited expandability of primary cells will hardly allow the production of clinically applicable MSC-EV products in scaled manners. Since EVs are not self-replicating, we considered the possibility to produce them from immortalized cell lines, which may overcome the aforementioned challenges related to primary MSCs as cell source for EVs. Therefore, we have set up a human telomerase reverse transcriptase (hTERT)-based immortalization strategy and raised immortalized MSCs at the clonal level to manufacture EV products with reproducible immunomodulatory in vitro activities (Mouloud et al., in preparation). However, in vitro immunomodulatory activities can hardly predict the therapeutic capacity of MSC-EVs in neurological disorders in vivo.

Our previous work in a murine model of neonatal HI-induced brain injury demonstrated that EVs from primary MSCs provide neuroprotection by regulating a combination of different processes, including inhibition of neuroinflammatory responses, promotion of regeneration, and support of important neurodevelopmental processes, i.e., oligodendrocyte maturation and myelination [14]. In addition to direct impacts on the brain, primary MSCs and their EV products were supposed to modulate peripheral immune responses in adult and neonatal brain injury models [8, 30, 31]. However, whether EVs from clonally expanded and immortalized MSCs (ciMSCs) retain their multimodal protective capacities in vivo remained unclear. Furthermore, in the majority of studies, MSC-EVs were administered intravenously or intraperitoneally [8, 12–15, 25–27, 32–34]. A less invasive intranasal route has been successfully established as an alternative administration mode for MSCs and MSC-EVs in various small and large animal models of perinatal brain injury and in a pilot clinical trial in neonatal stroke patients [10, 16, 35–41].

In the present study, we compared the therapeutic potential of intranasally administered EVs from primary and ciMSCs on HI-induced brain tissue loss. To get deeper insight into the underlying mechanisms of a ciMSC-EV therapy, we assessed neuroinflammatory, neuroregenerative, and neurodevelopmental processes.

## Methods

### Cultivation of MSCs and preparation of MSC-EVs

Primary MSCs and ciMSCs were raised and characterized as previously described [42]. Briefly, cells of given MSC stocks (MSC41.5, ciMSC41.5 clone 6) were expanded at 37 °C in a 5% CO_2_ atmosphere in DMEM low glucose (PAN Biotech, Germany), supplemented with 10% human platelet lysate (hPL), 100 U/ml penicillin–streptomycin-glutamine (Thermo Fisher Scientific, Germany), and 5 IU/ml heparin (Ratiopharm, Germany). In total, 2 × 10^7^ cells were seeded in Nunc EasyFill Cell Factory System (Thermo Fisher Scientific) and raised in 400-ml culture medium. As soon as MSCs reached a density of approximately 50% confluency, the culture media were exchanged, and conditioned media (CM) were harvested every 48 h until passaging, i.e., when MSCs reached 80–90% confluency. CM were cleared from residual cells and debris by 2000 × *g* centrifugation for 15 min (Rotor: JS-5.3; Beckman Coulter, Germany), and supernatants were stored at − 20 °C until further processing. CMs were screened regularly for mycoplasma contamination (VenorGeM OneStep, Minerva Biolabs, Germany). EVs were prepared from thawed pooled CMs according to our standard procedure, i.e., by polyethylene glycol 6000 (PEG) precipitation followed by ultracentrifugation [28, 29]. EVs were solved in 10-mM HEPES 0.9% NaCl buffer (Thermo Fisher Scientific) and stored at − 80 °C as 1-ml aliquots containing EVs harvested from CMs of 4 × 10^7^ MSCs. The batch of ciMSC-EV 41.5 clone 6 used in this study was prepared from a 1.2-l pool of CMs harvested from passages 34 to 37 corresponding to a total cell equivalence of 1.92 × 10^8^ cells. The primary MSC-EV 41.5 batch was prepared from a 4.4-l pool of passages 4 to 5 CMs corresponding to a cell equivalent of 6.45 × 10^8^ cells.

### MSC characterization

Primary MSCs as well as ciMSCs were analyzed according to the criteria of the *International Society of Cell and Gene Therapy* (ISCT) [43]. Morphology and the osteogenic and adipogenic differentiation potential of MSCs were analyzed as described previously [6, 42]. Results are shown in Supplementary Fig. 1 a and b. Cell surface phenotypes of EV-producing MSCs were analyzed by flow cytometry (CytoFLEX; Software CytExpert 2.3, Beckman-Coulter) following anti-CD14, anti-CD31, anti-CD34, anti-CD44, anti-CD45, anti-CD73, anti-CD90, anti-CD105, and anti-HLA-DR antibody staining (Suppl. Table 1, Suppl. Figure 1c).

### EV characterization

Obtained MSC-EV preparations were characterized according to the recommendation of *Minimal Information for Studies of Extracellular Vesicles 2018* (MISEV2018) criteria [44]. Results are presented in Supplementary Fig. 2 and Supplementary Table 2. Briefly, particle concentrations were measured by nanoparticle tracking analysis [45, 46] on a ZetaView platform (Particle Metrix, Germany). The protein concentration was assessed using the bicinchoninic acid (BCA) assay (Pierce, IL, USA) according to the manufacturer’s recommendations. Characterization of EV marker expression was performed as previously described [47, 48]. Briefly, 5 µl of PEG-UC-prepared EV samples was labelled with anti-CD9, anti-CD63, and anti-CD81 antibodies (Suppl. Table 1). Unstained samples and buffer without EVs but with antibodies were used as controls. All samples were incubated for 1 h in the dark at room temperature, followed by dilution in PBS (pH 7.4; Gibco) (100-fold for anti-CD9 and 40-fold for anti-CD63 or CD81). Samples were analyzed with an ImageStreamX Mark II instrument (Amnis/Luminex, USA). Laser and compensation settings are provided in Supplementary Tables 3 and 4. All data were acquired for 5 min at 60 × magnification and low flow rate (0.3795 ± 0.0003 μl/min). Data analyses were performed using IDEAS software version 6.2 as described previously [47, 48]. All fluorescent objects were plotted against the side scatter (Suppl. Figure 2). Images were analyzed for coincidences by using the spot counting feature. Events with multiple spots were excluded from further analysis.

For evaluation of the in vitro immunomodulatory function of MSC-EV products, 25-µg protein of EV preparations was tested in a multi-donor mixed lymphocyte reaction (mdMLR) assay as previously described [27, 49]. Briefly, after thawing, 6 × 10^5^ pooled peripheral blood mononuclear cells (PBMC) of 12 donors were seeded into each well of a 96-well u-bottom shape plate (Corning, Germany). Cells were cultured in the presence or absence of EV or control samples in 200-µl RPMI 1640 supplemented with 100 U/ml penicillin, 100 µg/ml streptomycin (all Thermo Fisher Scientific), and 10% human AB serum for 5 days at 37 °C in a 5% CO_2_ atmosphere. For analyses, cells were harvested and labelled with an antibody cocktail of anti-CD4, anti-CD8, anti-CD25, and anti-CD54 antibodies (Suppl. Table 1). Analyses were performed on a CytoFLEX flow cytometer (Software CytExpert 2.3, BeckmanCoulter). T cells were identified as CD4^+^ or CD8^+^ cells. Activated CD4 and CD8 cells were identified as CD25 and CD54 double-positive cells (Suppl. Figure 3).

### Animal care and allocation

Experiments were performed in accordance to the Animal Research Reporting of In Vivo Experiments (ARRIVE) guidelines with government approval by the State Agency for Nature, Environment and Consumer Protection North Rhine-Westphalia. C57BL/6 J mice were bred in house and kept under a 12-h light/dark cycle with food and water ad libitum. Bodyweight of pups was recorded at postnatal day 9 (P9), P10, P11, P12, P14, and P16. A total of 40 C57BL/6 mice (*n* = 24 males and *n* = 16 females) derived from 5 litters were enrolled. For all analyses, animals per litter and experiment were randomly assigned to 4 experimental groups: sham *n* = 8 (6 males, 2 females); HI + vehicle *n* = 10 (6 males, 4 females); HI + MSC-EV *n* = 11 (6 males, 5 females); and HI + ciMSC-EV *n* = 11 (6 males, 5 females) prior to intervention. To control the potential influence of weight and sex, a stratified randomization was performed followed by simple randomization within each block to assign pups to individual groups. Individuals involved in data analysis knew the animals’ designation but were blinded to group assignment. In total, 5 animals died (12.5%; 2 female HI + vehicle; 1 male and 1 female HI + MSC-EV; 1 male HI + ciMSC-EV). All animals died during hypoxia, i.e., after randomization and prior to vehicle/EV administration.

### Neonatal hypoxia–ischemia and ciMSC-EV treatment

Hypoxic-ischemic brain injury was induced in 9-day-old mice as previously described [10, 14, 50–52]. Briefly, the right common carotid artery was occluded through cauterization (high-temperature cauter, 1200 °C, Bovie, USA) under isoflurane anesthesia (1.5–4 Vol%) followed by 1-h hypoxia (10% O_2_) in an airtight oxygen chamber (OxyCycler, Biospherix, USA) after 1-h recovery with their dams. Animals were placed on a warming mat (Harvard Apparatus, USA) to maintain nesting temperature during hypoxia [51]. Sham animals were subjected to anesthesia and neck incision only. Perioperative analgesia was ensured by subcutaneous administration of 0.1 mg/kg buprenorphine. Aliquots of MSC-EV41.5 or ciMSC-EV41.5 clone 6 preparations (1 × 10^5^ cell equivalents/g bodyweight) were administered intranasally in 2 × 2.5 µl/g bodyweight per nostril. Vehicle-treated control animals received the same volume of 0.9% NaCl. Thirty minutes before EV/vehicle administration, 2.5 µl hyaluronidase (100 U, Sigma-Aldrich) was applied per nostril [9, 10]. According to our previous study, EVs or vehicle were administered at days 1, 3, and 5 post HI [14].

### Tissue preparation, histology, and immunohistochemistry

Seven days after HI, mice were deeply anesthetized with chloral hydrate and transcardially perfused with ice-cold PBS. Brains were removed and snap frozen on dry ice. Tissue injury was assessed on cresyl-violet-stained 20-µm cryostat sections. Tissue atrophy was determined by measurement of intact areas in ipsi- and contralateral hemispheres at a distance of 400 µm using ImageJ software (NIH, USA). Volumes were calculated for the total hemisphere and cortex between 1 and − 2.6 mm from bregma, for the striatum between 1 and − 0.6 mm from bregma, and for the hippocampus between − 1.0 and − 2.6 mm from bregma. Tissue loss was determined by comparison with contralateral volumes according to the following equation: 100 − (volume ratio (left vs. right)) × 100.

**Fig. 1:**
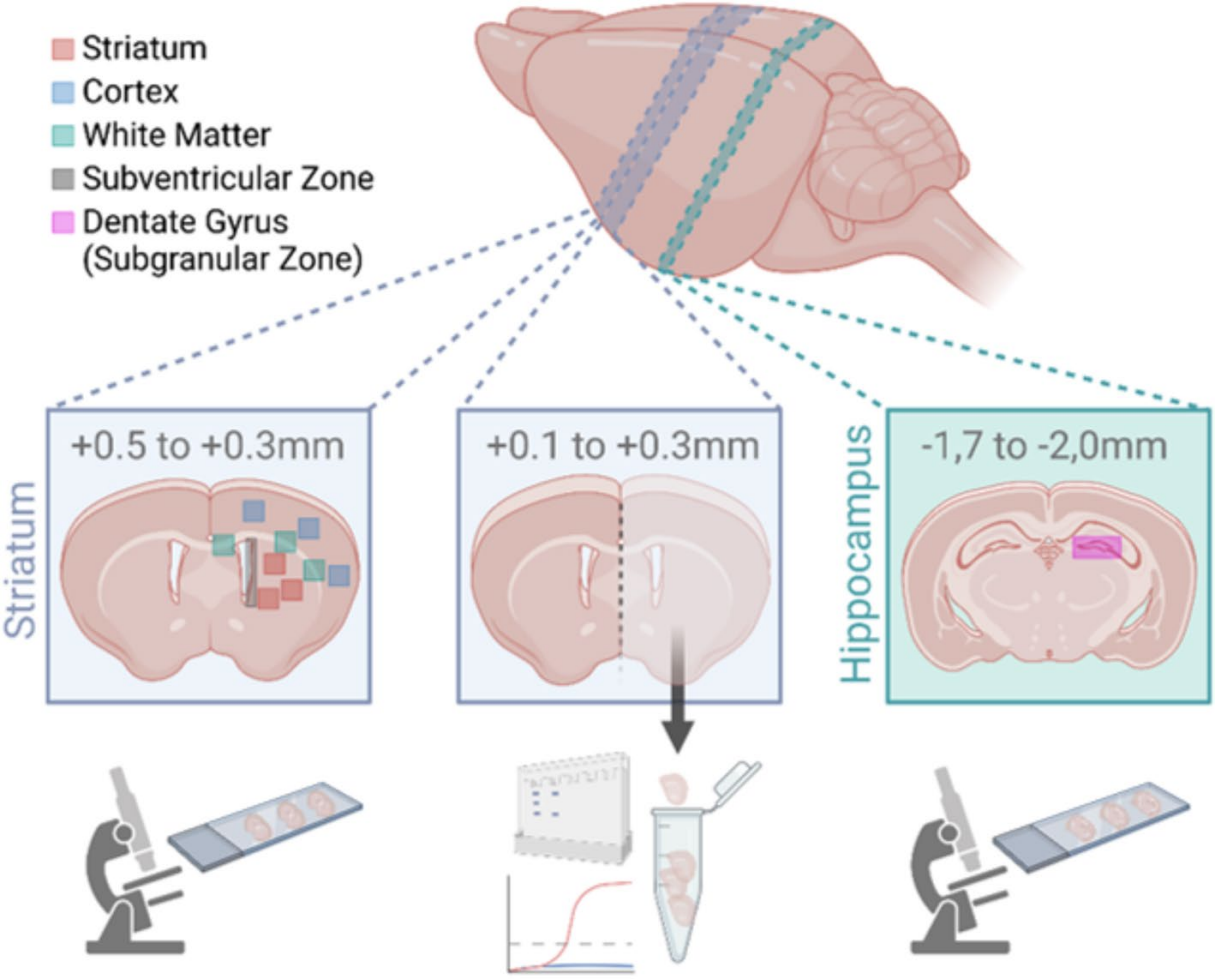
Overview about sample collection and brain regions analyzed by immunohistochemistry, western blot, and real-time PCR. For immunohistochemistry, 20-µm tissue sections were taken at the level of the striatum (bottom left) and the hippocampus (bottom right) at the indicated bregma levels. Regions of interest that were analyzed are marked in different colors according to the legend (top left). For western blot and real-time PCR analyses, 160-µm-thick tissue sections were prepared from the striatal level, and the total ipsilateral hemisphere of these sections was used for protein and RNA isolation (bottom middle). The figure was created with BioRender.com

For immunohistochemistry analyses, cryostat sections were taken at the level of 0.3 to 0.5 mm from bregma (striatal level) and − 1.7 to − 2.0 mm from bregma (hippocampal level) (Fig. 1). Tissue sections were stained as follows: For neuronal, oligodendrocyte, and vessel densities, neuronal nuclei (NeuN), oligodendrocyte transcription factor 2 (Olig2), and cluster of differentiation 31 (CD31) were stained, respectively. Proliferative responses, oligodendrocyte maturation, astrogliosis, and microglia were evaluated by staining of Ki67, adenomatous polyposis coli, clone CC1 positive (referred as CC1), glial fibrillary acidic protein (GFAP), and ionized calcium-binding adaptor protein-1 (Iba-1), respectively. Astrocytes were additionally characterized by co-staining of the pro-inflammatory A1 marker complement C3 (C3) [53]. Detailed information on primary and secondary antibodies is provided in Supplementary Table 5. Immunohistochemistry analyses were performed according to our previous studies [10, 14, 50, 51] with minor modifications. Briefly, tissue sections were thawed at 37 °C for 15 min followed by fixation in 4% paraformaldehyde (PFA) for NeuN, Olig2, Ki67 (host rat), CC1, and GFAP/C3 or in ice-cold acetone/methanol for CD31, Ki67 (host rabbit), CD45, and GFAP/Ki67 each for 5 min. For Iba-1/GFAP co-staining, sections were incubated with 4% PFA overnight at 4 °C followed by antigen retrieval in sodium citrate buffer (10-mM tri-sodium citrate, 0.05% Tween-20; pH 6.0) at 100 °C for 30 min. Unspecific antibody binding was blocked by incubation with 1% BSA, 0.3% cold fish skin gelatin (Sigma-Aldrich, Germany), and 0.2% Tween-20 in PBS for 1 h at room temperature followed by primary antibody incubation overnight at 4 °C. Antibody binding was visualized by incubation with appropriate anti-rat/mouse/rabbit Alexa Fluor 488-, Alexa Fluor 555-, or Alexa Fluor 647-conjugated secondary antibodies (all: 1:500, Thermo Scientific, Germany) for 1 h at room temperature. Nuclei were counterstained with 4′,6-diamidino-2-phenylindole (DAPI, 100 ng/ml; Molecular Probes, USA).

Confocal imaging (A1plus, Eclipse Ti, with NIS-Elements AR software, Nikon, Germany) was used to generate z-stack images (8 µm thickness, 2-µm focal plane distance) with a 20 × objective. Three nonoverlapping regions of interest (ROI, each 396.600 µm^2^) were acquired in the striatum and the cortex. Three additional ROIs were acquired in the white matter (i.e., corpus callosum) for assessment of oligodendrocyte proliferation and maturation. The exact localization of analyzed ROIs is presented in Fig. 1. For analyses of cellular proliferation in the subventricular zone (SVZ) and the subgranular zone (SGZ), large-scale images were acquired from 4 × 5 and 2 × 1 single images, respectively. Single images were processed to one image with the stitching tool of the NIS-Elements AR software. All images were converted into maximal intensity projections for automated software-based quantification using the NIS-Elements AR software. Unbiased software-based object counting was used to determine the number of NeuN^+^, Olig2^+^, Ki67^+^ cells, and CD31^+^ vessels. Single object counting was not possible for Iba-1 and GFAP staining due to intensive local accumulation of microglia and glial scar formation by astrocytes in severely injured regions and animals. Therefore, positively stained areas were quantified as a measure of cell density. According to previous reports, showing that not only the number and soma size of microglia but also the expression level of Iba-1 per cell increases following activation [54], we additionally quantified the staining intensity (i.e., mean fluorescence intensity) on Iba-1 positively labelled areas. To determine the expression level of the astrocytic A1 marker C3, we quantified mean fluorescence intensities of C3 immunostaining on GFAP^+^ areas. For assessment of oligodendrocyte maturation and proliferation, Olig2/CC1 and Olig2/Ki67 double-positive cells were counted manually. Astrocyte proliferation was analyzed in higher magnification images (5 µm thickness, 1-µm focal plane distance) acquired with the 40 × objective in the cortex and striatum (3 ROIs in each region). GFAP/Ki67 double-positive cells were counted manually.

### RNA and protein isolation

For RNA and protein isolation, 160-µm-thick tissue sections were collected at the striatal level (0.1 to 0.3 mm from bregma, Fig. 1). From these tissue sections, ipsilateral hemispheres were used to isolate total RNA and proteins via the TRIzol procedure (Thermo Fisher, Germany) using the QIAzol Reagent (Qiagen, Germany). Briefly, tissue samples were homogenized in QIAzol Reagent followed by the addition of chloroform to separate the homogenate into different phases. The clear upper aqueous phase was used for RNA precipitation. The precipitated RNA was washed with ethanol, subsequently air-dried, and resuspended in RNAse-free water. RNA yield was determined with the NanoDrop Spectrophotometer (Peqlab, Germany). To pellet DNA of the interphase, ethanol was added to the phenol–chloroform phase and centrifuged. The supernatant was used for protein precipitation by adding isopropanol, and the DNA pellet was discarded. Precipitated protein samples were washed three times with guanidine hydrochloride, followed by an ethanol washing step and air-drying of the pellet. Pellets were dissolved in 1% sodium dodecyl sulfate (SDS) for 2 days at 40 °C.

### mRNA expression analysis

For mRNA expression analysis, 1.2 µg of total RNA and TaqMan reverse transcription reagents (Applied Biosystems/Thermo Fisher Scientific) were used to synthesize first-strand complementary DNA. Real-time polymerase chain reaction (PCR) was performed in duplicates in 96-well-optical reaction plates for 40 cycles with each cycle at 94 °C for 15 s and 60 °C for 1 min using the StepOnePlus Real-Time PCR system (Applied Biosystems/Thermo Fisher Scientific). PCR products were quantified using assay on demand primers and fluorogenic reporter oligonucleotide probes (Applied Biosystems/Thermo Fisher Scientific, Suppl. Table 6). Ct values were normalized to the housekeeping gene beta-2-microglobulin [*Δ*ct = ct (target gene) — ct (beta-2-microglobulin)] and related to the mean of sham animals using the *ΔΔ*CT formula [*ΔΔ*CT = *Δ*CT (sham) — *Δ*CT (MSC-EV)]. Fold change values were calculated.

### Western blot analysis

After quantification of the protein concentration using the Pierce BCA assay (Thermo Scientific, USA), protein lysates were separated on 12.5% SDS polyacrylamide gels and transferred to nitrocellulose membranes (0.2 µm, Amersham, USA) at 4 °C overnight. Equal loading of 7.5-µg protein per lane and transfer of proteins were confirmed by staining of membranes with Ponceau S solution (Sigma Aldrich). Nonspecific binding was blocked by incubation in 5% nonfat milk powder (Cell Signaling, USA) and 0.1% Tween-20 in tris-buffered saline (TBS) followed by incubation with the primary antibodies. The following primary antibodies were applied: rabbit anti-NeuN, goat anti-vascular cell adhesion molecule-1 (VCAM-1), rabbit anti-Iba-1, mouse anti-GFAP, and rabbit anti-glutaraldehyde-3-phosphate dehydrogenase (GAPDH) (Suppl. Table 7), each in blocking solution at 4 °C overnight. Membranes were incubated with appropriate peroxidase-conjugated secondary antibodies (all 1:5000, Dako, Denmark) in blocking solution at room temperature for 1 h followed by chemiluminescent detection with the enhanced chemiluminescence prime Western blotting detection reagent (Amersham, GE Healthcare Life Science, USA). For visualization and densitometric analysis, the ChemiDocXRS + imaging system and ImageLab software (Bio-Rad, Germany) were used.

### Assessment of inflammatory mediators in serum samples

Blood was collected from the right atrium prior to perfusion and collected in ethylenediaminetetraacetic acid-coated tubes. Samples were centrifuged at 2000 × *g* for 10 min at 4 °C. The supernatant was frozen at − 80 °C until further analysis. Serum samples were analyzed for the abundance of TNF-alpha, IL-6, IL-17, IL-15, IL-10, and MMP9 performing an MSD multiplex screening assay (Meso Scale Discovery, USA) according to the manufacturers’ instructions. Per assay, a minimal amount of 25-µl serum was needed for analyses. One animal of sham-operated mice had to be excluded from these analyses due to a too small amount of blood (i.e., serum) that could be collected. Samples were analyzed with the MESO QuickPlex SO 120MM system using the Methodical Mind software (Meso Scale Discovery, USA).

### Statistical analysis

All results are expressed as box plots with individual data points including median values, the 25% and the 75% percentile. For statistical analysis, the GraphPad Prism 9.0 software package (GraphPad Software) was used. Data were tested for Gaussian distribution and analyzed either by ordinal one-way ANOVA or by Kruskal–Wallis (non-parametric) with post hoc Sidak’s or Dunn’s multiple comparison tests, respectively.

## Results

### Intranasal delivery of ciMSC-EVs provides similar neuroprotection as EVs from primary MSCs

**Fig. 2:**
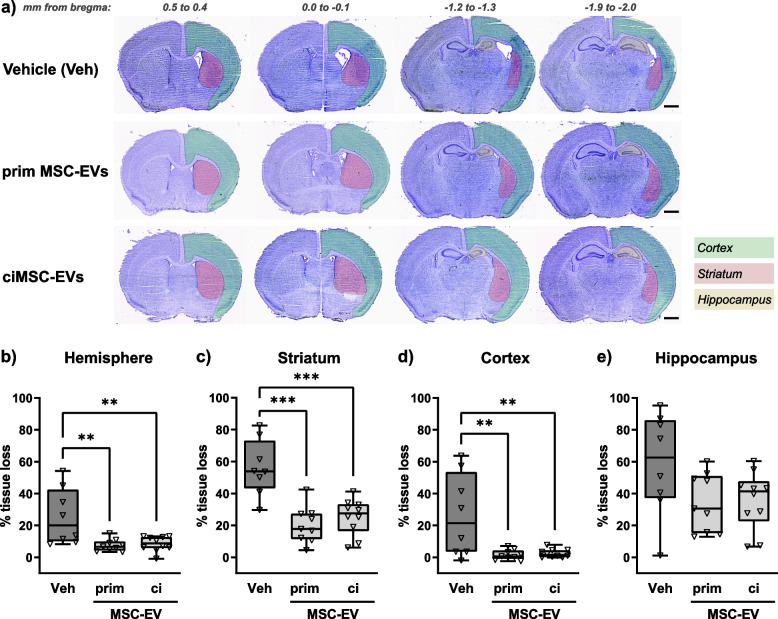
Intranasal ciMSC-EV delivery provides similar protection from HI-induced tissue loss as EVs from primary MSCs. Postnatal day 9 (P9) C57BL/6 mice were exposed to HI followed by i.n. administration of vehicle (0.9% NaCl, Veh), EVs from primary MSCs (prim), or ciMSC-EVs 1, 3, and 5 days after HI. At P16, histological brain injury was assessed on cresyl violet-stained 20-µm cryostat sections (**a**, scale bar: 1 mm). Coronal tissue sections at the indicated bregma levels are presented in **a** to reveal tissue loss in different regions according to the legend provided. Quantification of brain atrophy was performed by measurement of intact areas on tissue sections at a distance of 400 µm between + 1 and − 2.6 mm from bregma. Volumes were calculated for total hemispheres (**b**), striatum (**c**), cortex (**d**), and hippocampus (**e**). Tissue loss is expressed as the percentage of volume reduction compared to intact contralateral volumes. **p < 0.01, ***p < 0.0001, *n* = 8–10/group

In the present study, we chose a low invasive delivery route by intranasal (i.n.) application, previously suggested to result in a more efficient uptake of different neurotherapeutics compared to intraperitoneal (i.p.) injection [55]. To allow retrospective comparisons between different delivery routes, in the present injury model, the same time points and dosing of applied EV preparations were used as in our previous work with i.p. administration [14]. The impact of i.n.-delivered EVs derived from primary MSCs and ciMSCs on HI-induced brain tissue loss was analyzed in cresyl-violet-stained tissue sections. Exemplary images of prominent brain levels are presented in Fig. 2a, to demonstrate tissue loss from caudal to rostral brain levels. Quantification of regional and total hemisphere volume loss revealed comparable neuroprotective effects of primary MSC-EVs and ciMSC-EVs for the total hemisphere (Fig. 2 a, b). Furthermore, i.n. administration of EVs from both cell sources significantly reduced HI-induced tissue atrophy by 50–70% in the striatum (Fig. 2c) and by 90–95% in the cortex (Fig. 2 a, d), while effects on the hippocampus were less pronounced with a reduction by 35–50% (Fig. 2 a, e). Because of the comparable therapeutic potency of both EV products, we focused our more detailed analyses on effects of i.n.-delivered ciMSC-EVs on the most protected brain regions, i.e., the striatum and the cortex.

### ciMSC-EV treatment protects from HI-induced neuronal loss and impaired neurogenic proliferation

**Fig. 3:**
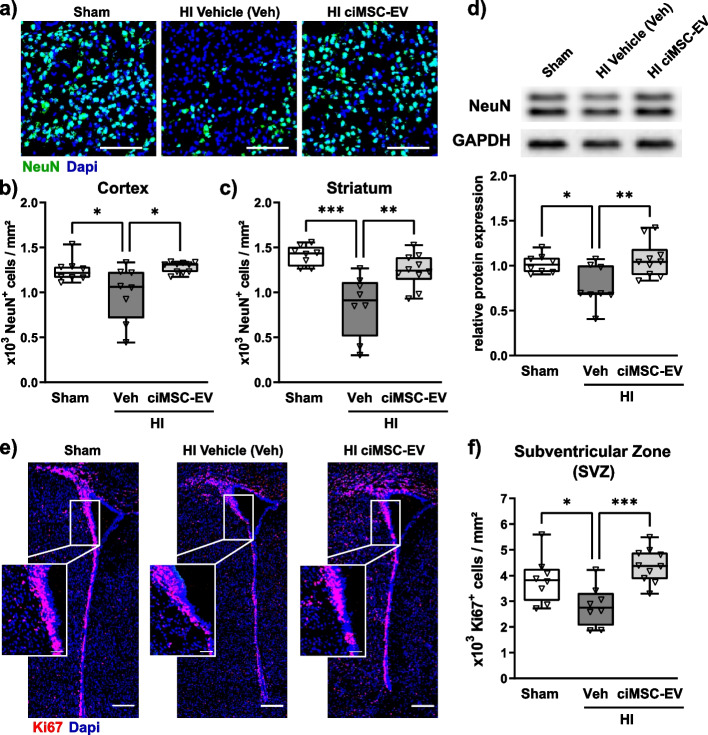
ciMSC-EVs protect from HI-induced neuronal loss and increase cellular proliferation in the neurogenic subventricular zone. Neuronal density was analyzed via immunohistochemistry for NeuN (**a**) in 16-day-old C57BL/6 mice that were exposed to HI on postnatal day 9 followed by i.n. delivery of 0.9% NaCl (vehicle, Veh) or ciMSC-EVs 1, 3, and 5 days after HI. Immunohistochemistry analyses for NeuN were performed in cortex (**b**) and striatum (**c**). From the same animals, protein lysates from the entire hemisphere derived from 160-µm-thick tissue sections at the level of striatum were prepared and analyzed via western blot (**d**). Data were normalized to the reference protein GAPDH and to sham animals (**d**). The number of proliferating cells was determined via immunohistochemistry for the proliferation marker Ki67 in the subventricular zone (SVZ, **e**, **f**). Representative images in **a** are derived from the striatum (scale bar: 100 µm). Low magnification images in **e** show the total SVZ (scale bar: 200 µm); insets reveal higher magnification pictures (scale bar: 50 µm) of rectangles depicted in low magnification images. Representative western blot images in **d** show examples of protein abundance in tissue lysates obtained from sham-operated, HI-injured vehicle-treated, and HI-injured ciMSC-EV-treated animals. Images were cropped and scaled for illustration purposes from original western blot images provided in Suppl. Figure 6. *p < 0.05, **p < 0.01, ***p < 0.001, *n* = 8–10/group

To identify processes being affected by intranasal ciMSC-EV treatment, we quantified the impact on HI-induced neuronal loss, assessed via immunohistochemistry for the neuronal marker NeuN (Fig. 3a) in the cortex (Fig. 3 a, b) and striatum (Fig. 3c). Compared to healthy sham-operated control mice, we detected a pronounced reduction of NeuN-positive cells in both regions of vehicle-treated but not ciMSC-EV-treated HI-injured animals (Fig. 3 a–c). Similarly, western blot analyses of tissue lysates from tissue 160-µm-thick tissue sections of the entire hemisphere at the striatal level demonstrated a significantly reduced NeuN expression in vehicle-treated HI-injured animals compared to sham-operated and ciMSC-EV-treated HI animals (Fig. 3d).

To assess whether effects of an ciMSC-EV treatment were mainly attributed to prevention of neuronal cell loss and consecutive brain atrophy or may be also related to restauration of impaired cellular proliferation, we quantified the amount of Ki67-positive cells (Fig. 3, Suppl. Figure 4). Compared to sham-operated animals, we detected more Ki67-positive cells in the cortex and striatum of HI-injured animals, which was independent of a ciMSC-EV treatment (Suppl. Figure 4 a, b). In contrast, in the neurogenic zones of the CNS, i.e., the subventricular zone (SVZ) and the subgranular zone (SGZ), HI induced a strong reduction of Ki67-positive cells (Fig. 3 e, f; Suppl. Figure 4c). Notably, ciMSC-EV treatment completely restored HI-induced impairment of cellular proliferation in the SVZ (Fig. 3 e, f), while only a slight improvement, not reaching statistical significance, was observed in the SGZ (Suppl. Figure 4c).

### ciMSC-EV application prevents HI-induced vascular injury, counteracts impaired endothelial cell proliferation, and decreases endothelial activation as well as leukocyte infiltration

**Fig. 4:**
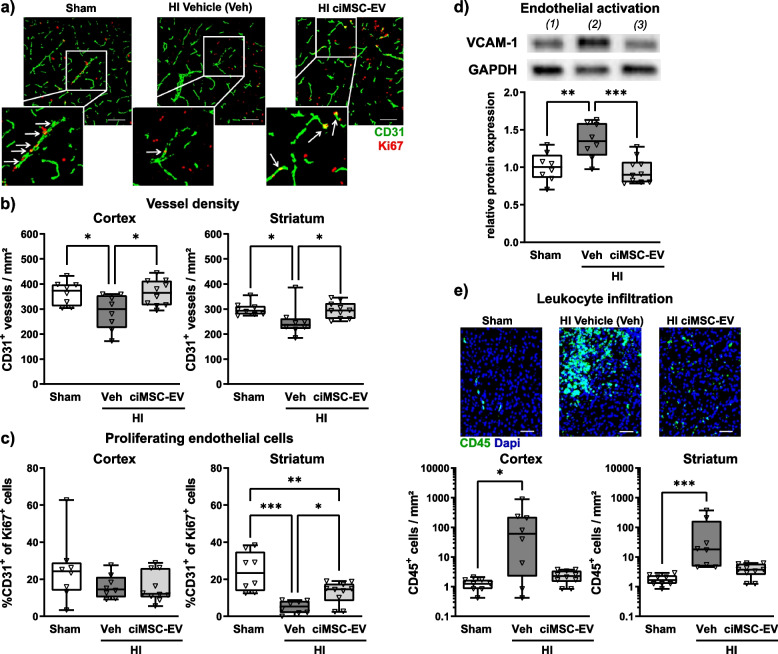
ciMSC-EVs prevent HI-induced vessel loss and impaired endothelial proliferation while reducing endothelial activation and leukocyte infiltration. Postnatal day 9 (P9) C57BL/6 mice were exposed to HI followed by i.n. administration of vehicle (0.9% NaCl, Veh) or ciMSC-EVs 1, 3, and 5 days after HI. At P16, vessel density and endothelial proliferation were quantified via immunohistochemistry for the pan-vessel/endothelial cell marker CD31 and the proliferation marker Ki67 (**a**) in the cortex (**b**) and striatum (**c**). Vessel densities were quantified by unbiased automated software-based object detection (**b**). Endothelial cell proliferation was determined by counting CD31/Ki67 double-positive cells (**c**). Endothelial activation was determined by western blot analysis for the adhesion molecule VCAM-1 (**d**). Data were normalized to the reference protein GAPDH and to sham-operated mice (**d**). Leukocyte infiltration was analyzed via immunohistochemistry for the pan-leukocyte marker CD45 (**e**). Representative images in **a** and **e** are derived from the striatum (scale bar in **a**: 100 µm, scale bar in **e**: 50 µm). Representative western blot images in **d** show examples of protein abundance in tissue lysates obtained from *(1)* sham-operated, *(2)* HI-injured vehicle-treated, and *(3)* HI-injured ciMSC-EV-treated animals. Images were cropped and scaled for illustration purposes from original western blot images provided in Suppl. Figure 6. *p < 0.05, **p < 0.01, ***p < 0.001, *n* = 8–10/group

Endothelial cell proliferation and vascularization are important neurodevelopmental processes, which are impaired by HI [14, 56]. Therefore, we analyzed the vascular density and quantified the number of proliferating endothelial cells via immunohistochemistry for the endothelial cell marker CD31 and the proliferation marker Ki67. Intranasal delivery of ciMSC-EVs protected from HI-induced reduction of CD31^+^ vessels in the cortex and striatum (Fig. 4 a, b). Of note, this effect was accompanied by a significantly enhanced proportion of CD31/Ki67 double-positive cells in the striatum of ciMSC-EV-treated HI-injured animals compared to vehicle-treated HI animals, though not reaching levels of sham mice (Fig. 4c). In addition to vascular injury and disturbed endothelial proliferation, HI induces endothelial activation, characterized by an increased expression of adhesion molecules, like VCAM-1, facilitating infiltration of peripheral leukocytes into the injured brain [10, 57, 58]. Western blot analyses of lysed brain sections revealed an upregulation of VCAM-1 in vehicle-treated HI animals, while ciMSC-EV treatment resulted in VCAM-1 protein abundance comparable to sham-operated mice (Fig. 4d). Reduced VCAM-1 expression in ciMSC-EV-treated animals coincided with a less pronounced HI-induced infiltration of peripheral leukocytes, as demonstrated by a decreased accumulation of CD45^+^ cells (Fig. 4e).

### ciMSC-EV treatment attenuates HI-induced microglia activation, associated with an increased expression of anti-inflammatory cytokines in the brain

**Fig. 5:**
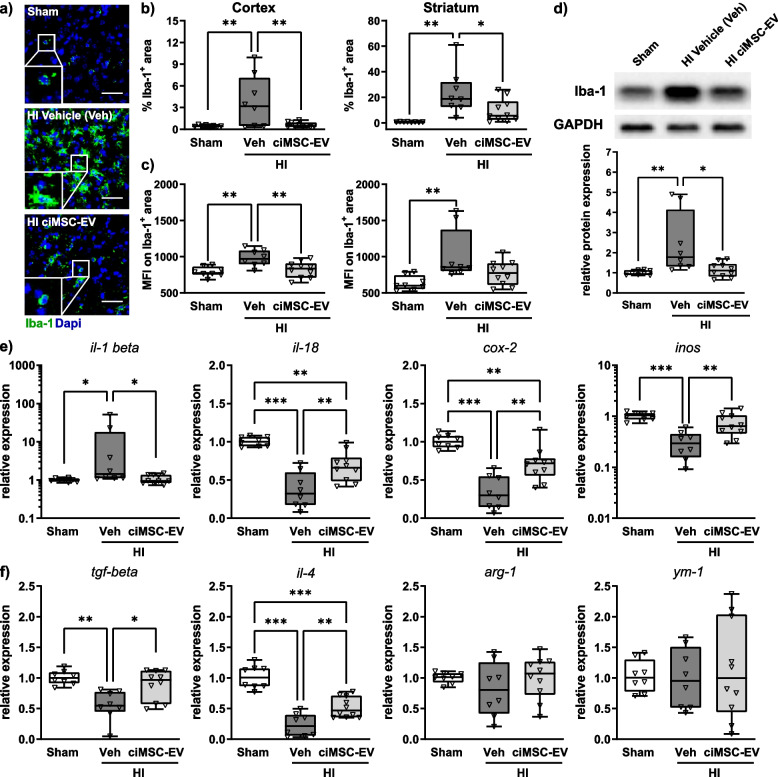
ciMSC-EV treatment decreases HI-induced microglia activation, associated with alterations of typical M1/M2 marker expression. Postnatal day 9 (P9) C57BL/6 mice were exposed to HI followed by i.n. administration of vehicle (0.9% NaCl, Veh) or ciMSC-EVs 1, 3, and 5 days after HI. Analyses were performed at P16. Microglia density and activation were analyzed by quantification of Iba-1 immunoreactivity via immunohistochemistry (**a**–**c**) and western blot (**d**). Iba-1 immunoreactivity, as a measure of microglia activation, was quantified by measurement of positively stained areas (**b**) and mean fluorescence intensities (MFI) on Iba-1-positive areas (**c**) in the cortex and striatum. For western blot analyses, data of Iba-1 were normalized to the reference protein GAPDH and to sham-operated animals (**d**). A broad set of pro-inflammatory M1-phenotype-associated (**e**) and anti-inflammatory M2-phenotype-associated (**f**) molecules was analyzed via real-time PCR in brain tissues obtained from 160-µm-thick tissue sections at the striatal level. Beta-2-microglobulin served as housekeeping gene, and fold change values compared to sham animals were calculated (**e**, **f**). Representative images in **a** are derived from the striatum (scale bar: 50 µm). Higher magnification images of single cells derived from depicted rectangles in low magnification images are provided in insets to demonstrate morphological changes exemplarily (**a**). Representative western blot images in **d** show examples of protein abundance in tissue lysates obtained from sham-operated, HI-injured vehicle-treated, and HI-injured ciMSC-EV-treated animals. Images were cropped and scaled for illustration purposes from original western blot images provided in Suppl. Figure 6. *p < 0.05, **p < 0.01, ***p < 0.001, *n* = 8–10/group

In addition to the infiltration of peripheral immune cells, neonatal HI induces a strong local inflammatory response, characterized by an increased microglia accumulation and activation [59]. Since dense accumulation of microglia in severely affected HI-injured animals challenges single-cell counting (Suppl. Figure 5), we quantified the positively labelled Iba-1 area, demonstrating a strong upregulation in the cortex and striatum of vehicle- but not ciMSC-EV-treated HI-injured mice (Fig. 5 a, b). Qualitative assessment of morphological changes showed a hypertrophic and hyperramified phenotype in vehicle-treated HI animals, while microglia of ciMSC-EV-treated mice revealed a morphology comparable to that of healthy sham-operated animals (Fig. 5a, insets). In addition to increased cell densities and morphological changes, recent reports suggested that microglia activation is associated with an increased Iba-1 expression per microglia cell [54, 60]. Quantification of the fluorescence signal intensity on Iba-1 positively stained areas demonstrated a significant increase in the cortex and striatum of vehicle- but not ciMSC-EV-treated HI-injured mice (Fig. 5c). Results obtained from regional analyses via immunohistochemistry were confirmed by western blot analyses in the entire hemisphere at the level of the striatum (Fig. 5d).

Microglia can acquire different activation states contributing to both brain damage and repair [61]. Being aware about the difficulty of terminology [62], we refer to the commonly used M1/M2 nomenclature, with M1 cells supposed to mediate pro-inflammatory/neurotoxic effects and M2 cells suggested to have anti-inflammatory and regenerative functions [61]. To study potential impacts of a ciMSC-EV treatment on microglia polarization, we quantified the expression of several M1- (Fig. 5e) and M2 (Fig. 5f)-related molecules via real-time PCR. In accordance to our previous study [14], HI induced a strong upregulation of the pro-inflammatory cytokine IL-1-beta and a downregulation of IL-18, Cox-2, and iNos (Fig. 5e). These effects were counteracted by ciMSC-EV treatment (Fig. 5e). Consistently, HI-induced reduction of mRNA expression encoding for the anti-inflammatory cytokines TGF-beta and IL-4 was less pronounced in ciMSC-EV- than in vehicle-treated animals (Fig. 5f). mRNA expression of the M2 markers Arg-1 and YM-1 was not modulated, neither by HI nor ciMSC-EV treatment (Fig. 5f).

### Intranasal ciMSC-EV application does not modulate the level of circulating inflammatory mediators

**Fig. 6:**
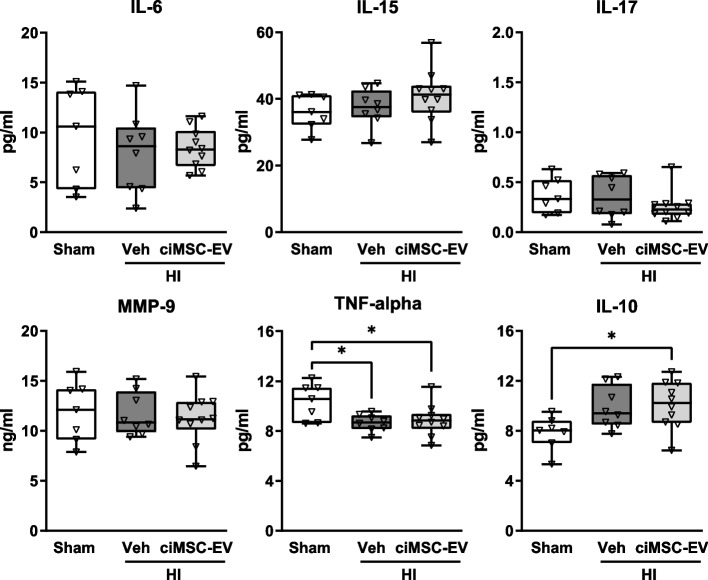
Intranasal ciMSC-EV delivery does not alter the level of inflammatory mediators in the peripheral blood. Postnatal day 9 (P9) C57BL/6 mice were exposed to HI followed by i.n. administration of vehicle (0.9% NaCl, Veh) or ciMSC-EVs 1, 3, and 5 days after HI. At P16, serum samples were collected and analyzed for the abundance of different inflammatory mediators, as depicted in the figure. *p < 0.05, *n* = 7–10/group

While the present findings provide clear evidence that i.n. delivery of ciMSC-EVs has strong immunomodulatory and neuroprotective effects in the brain, alteration of peripheral immune responses may have contributed to the overall improved outcome, too. Therefore, we analyzed the protein concentration of different pro- and anti-inflammatory mediators in the peripheral blood. While no differences were observed for IL-6, IL-15, IL-17, and MMP9, HI induced a significant reduction of TNF alpha and an increase of IL-10, which was, however, not altered in ciMSC-EV-treated animals (Fig. 6).

### ciMSC-EV treatment decreases astrogliosis and enhances growth factor expression

**Fig. 7:**
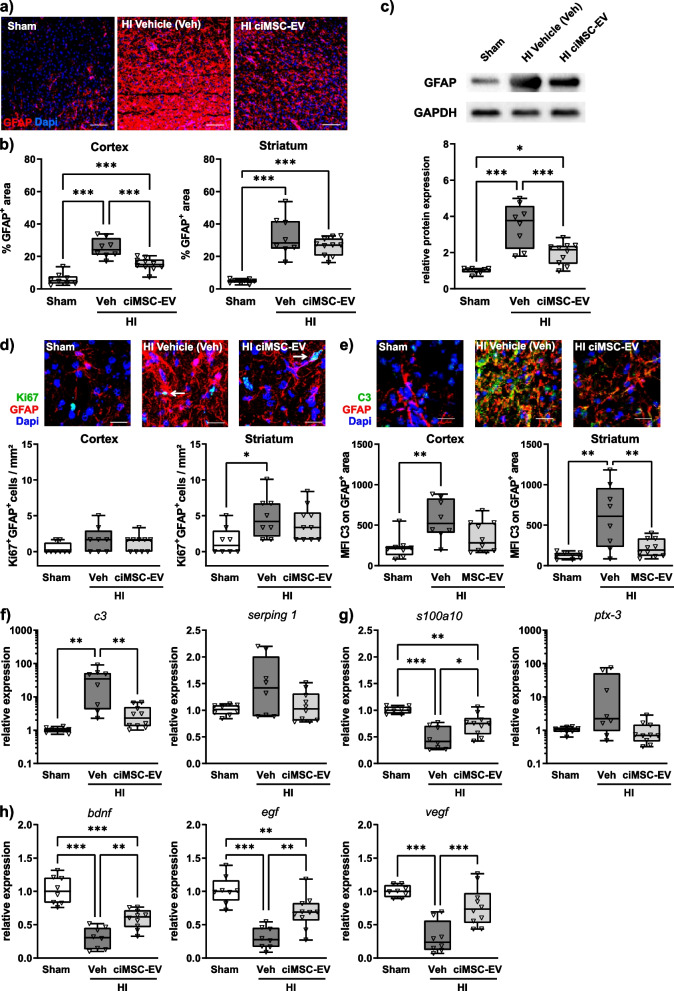
ciMSC-EVs protect from HI-induced astrocyte activation, associated with an increased expression of neural growth factors. Postnatal day 9 (P9) C57BL/6 mice were exposed to HI followed by i.n. administration of vehicle (0.9% NaCl, Veh) or ciMSC-EVs 1, 3, and 5 days after HI. Analyses were performed at P16. Astrocyte density and activation were analyzed by immunohistochemistry (**a**, **b**) and western blot (**c**) for GFAP. GFAP immunoreactivity, as a measure of reactive astrocytes, was quantified by measurement of positively stained areas in the cortex and striatum (**b**). For western blot analyses, lysates of entire ipsilateral hemispheres derived from 160-µm-thick tissue sections at the striatal level were analyzed (**c**). Data of GFAP were normalized to the reference protein GAPDH and to sham-operated animals (**c**). Proliferating astrocytes were quantified in tissue sections stained for Ki67 and GFAP (**d**). Double-positive cells were quantified in the cortex and striatum (**d**). Expression of the typical pro-inflammatory/neurotoxic A1-phenotype-associated molecule complement 3 (C3) was evaluated by measurement of mean fluorescence intensities (MFI) of C3 staining on GFAP positively labelled areas (**e**). Additional markers associated with pro-inflammatory/neurotoxic A1 (**f**) or anti-inflammatory/regenerative A2 astrocytes (**g**) and essential neural growth factors (**h**) were analyzed via real-time PCR in brain tissues, obtained from 160-µm-thick tissue sections from the striatal level. Beta-2-microglobulin served as housekeeping gene, and fold change values compared to sham animals were calculated (**f**–**h**). Representative immunofluorescence images are derived from the cortex in **a** and **e** and from the striatum in **d**. Scale bar in **a**: 100 µm, scale bar in **d** and **e**: 20 µm. Arrows in representative images of **d** indicate GFAP/Ki67 double-positive cells. Representative western blot images in **c** show examples of protein abundance in tissue lysates obtained from sham-operated, HI-injured vehicle-treated, and HI-injured ciMSC-EV-treated animals. Images were cropped and scaled for illustration purposes from original western blot images provided in Suppl. Figure 6. *p < 0.05, **p < 0.01, ***p < 0.001, *n* = 8–10/group

After showing that an intranasal delivery of ciMSC-EVs does not modulate peripheral immune responses, we further focused on neuroinflammation in the brain. A major hallmark of HI-induced neuroinflammation is astrogliosis. Following an injury, astrocytes proliferate and enlarge their projections, which can be measured by an increased GFAP expression [63]. As expected, HI induced a strong upregulation of GFAP in the cortex and striatum and in tissue lysates of the total hemisphere (Fig. 7 a–c). Intranasal application of ciMSC-EVs resulted in a significantly decreased GFAP expression in the cortex (Fig. 7 a, b) and in tissue lysates of the total hemisphere (Fig. 7c). To investigate whether these differences in GFAP immunoreactivity were related to alteration of astrocyte proliferation, a hallmark of reactive astrocytes [63–65], we quantified the number of Ki67^+^ astrocytes (Fig. 7d). HI induced a significant increase of proliferating astrocytes in the striatum, which was not modulated by i.n. delivery of ciMSC-EVs (Fig. 7d). In the cortex, neither HI nor ciMSC-EV treatment significantly modulated the amount of GFAP/Ki67 double-positive cells. However, it should be noted that the number of these cells was small in both regions (Fig. 7d). According to the increasingly acknowledged concept that reactive astrocytes represent a diverse cell population, also including non-proliferating cells [63–65], we aimed to characterize phenotypical changes of astrocytes in more detail. Similar to microglia, astrocytes are supposed to obtain different activation states, with A1 cells suggested to mediate pro-inflammatory and degenerative effects, and A2 cells supposed to exert anti-inflammatory and pro-regenerative effects [53]. Immunohistochemistry analyses of astrocytic C3 expression, recently suggested to be upregulated on pro-inflammatory A1 cells [53], demonstrated a strong increase in vehicle-treated HI-injured animals, which was prevented by i.n. delivery of ciMSC-EVs (Fig. 7e). These findings were consolidated by results of mRNA expression analyses, investigating expression of typical A1- and A2-associated molecules in tissue lysates of the entire hemisphere at the striatal level (Fig. 7 f, g). Confirming results from immunohistochemistry, we detected a strong upregulation of C3 expression in vehicle-treated HI-injured animals, which was significantly attenuated in ciMSC-EV-treated HI mice (Fig. 7f). Furthermore, HI led to a downregulation of the A2 marker S100A10, which was significantly increased after ciMSC-EV application (Fig. 7g). No significant group differences were detected for the A1 marker Serping 1 (Fig. 7f) and the A2 marker Pentraxin 3 (PTX3, Fig. 7g). In addition to modulation of neuroinflammatory responses, astrocytes are a major source of trophic growth factors, which are important for neurorepair [66]. Real-time PCR analyses showed that neonatal HI induces a strong reduction of BDNF, EGF, and VEGF mRNA expression; this effect was significantly attenuated in ciMSC-EV-treated animals (Fig. 7h).

### ciMSC-EV application restores HI-induced deficits in oligodendrocyte maturation

**Fig. 8:**
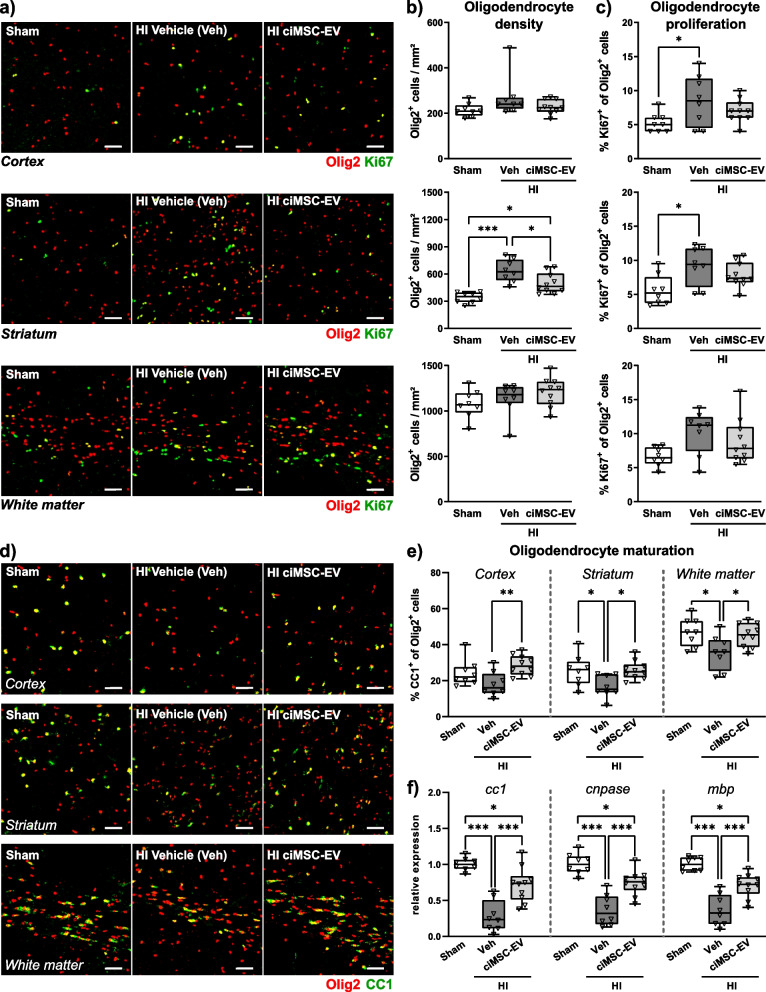
ciMSC-EV treatment attenuates HI-induced deficits in oligodendrocyte maturation. Postnatal day 9 (P9) C57BL/6 mice were exposed to HI followed by i.n. administration of vehicle (0.9% NaCl, Veh) or ciMSC-EVs 1, 3, and 5 days after HI. Analyses were performed at P16. Oligodendrocyte density (**a**, **b**), oligodendrocyte proliferation (**a**, **c**), and oligodendrocyte maturation (**d**, **e**) were investigated in the cortex, striatum, and white matter via immunohistochemistry for the pan-oligodendrocyte marker Olig2 combined either with the proliferation marker Ki67 (**a**) or CC1, labeling mature oligodendrocytes (**d**). The amount of Olig2 single (**b**) and Olig2/Ki67 (**c**) or Olig2/CC1 (**e**) double-positive cells was quantified. mRNA expression analyses were performed for markers expressed by mature and myelinating oligodendrocytes in brain tissue lysates obtained from 160-µm-thick tissue sections at the striatal level (**f**). Beta-2 microglobulin served as housekeeping gene, and fold change values compared to sham animals were calculated (**f**). Representative images in **a** and **d** are derived from the indicated brain regions (scale bar: 50 µm). Examples for the white matter are obtained from the corpus callosum. *p < 0.05, **p < 0.01, ***p < 0.001, *n* = 8–10/group

While HI-induced inflammatory responses contribute to secondary neurodegeneration, HI also induces endogenous regenerative responses like oligodendrogenesis [10, 51]. However, important neurodevelopmental processes like oligodendrocyte maturation, lasting into early adulthood, are impaired by HI [14]. To assess oligodendrocyte proliferation and maturation, immunohistochemistry analyses for the pan-oligodendrocyte marker Olig2 were combined with staining for either Ki67 (proliferation, Fig. 8 a–c) or CC1, a marker for mature oligodendrocytes (Fig. 8 d, e). In addition to the cortex and striatum, we also analyzed structures of the white matter, a major site of oligodendrocyte proliferation and maturation. Quantification of the total amount of Olig2-positive cells demonstrated a significant increase in the striatum of HI-injured animals compared to healthy sham-operated mice (Fig. 8 a, b), which was associated with an increased proportion of proliferating (i.e., Olig2/Ki67 double positive) oligodendrocytes (Fig. 8c). A similar increase in the percentage of proliferating oligodendrocytes was observed in the cortex and the white matter, though not reaching significance in the latter region (Fig. 8c). Except of a slightly reduced total amount of oligodendrocytes in the striatum of ciMSC-EV-treated HI-injured animals (Fig. 8 a, b), the percentage of proliferating cells was similar between vehicle- and ciMSC-EV-treated mice in all analyzed regions (Fig. 8c). However, in spite of an increased proliferative response following HI (Fig. 8 a, c), the proportion of mature oligodendrocytes was significantly reduced in the striatum and white matter of vehicle-treated HI-injured animals compared to healthy sham-operated mice (Fig. 8 d, e). This effect was attenuated by intranasal ciMSC-EV administration (Fig. 8e). To confirm effects on oligodendrocyte maturation, we quantified mRNA expression of CC1 and of the myelin proteins CNPase and MBP in tissue lysates of the total hemisphere (Fig. 8f). In accordance to results from immunohistochemistry analyses, HI induced a strong downregulation of all analyzed molecules. The severity of this detrimental HI effect was significantly reduced upon ciMSC-EV treatment (Fig. 8f).

## Discussion

In spite of the growing body of evidence for the neuroprotective potential of MSC-EVs in perinatal brain injury [12, 14–16, 40], major challenges with regard to clinical translation are MSC senescence and MSC heterogeneity. To overcome these limitations, we have immortalized primary human bone marrow-derived MSCs and expanded them at the clonal level. In the present study, we demonstrate that EVs manufactured from these ciMSCs are still potent in suppressing HI-induced brain injury at different levels. Following i.n. administration, ciMSC-EVs reduced microglia activation, astrogliosis, endothelial activation, and leukocyte infiltration. These anti-inflammatory effects were associated with an improvement of neuroregenerative and neurodevelopmental processes, including neural progenitor and endothelial cell proliferation, oligodendrocyte maturation, and neurotrophic growth factor expression. These findings reveal that the applied immortalization strategy does not affect the neuroprotective properties of EVs from primary MSCs.

We administered MSC-EVs intranasally since this route is less invasive than i.p. or i.v. injections and appears beneficial for the treatment of perinatal brain injuries with MSC products [16, 36, 40, 67–69]. Comparing the present findings with results from our recent work, in which EVs from the same MSC stock (MSC 41.5) were administered via i.p. injections [14], we even detected a clear advantage of i.n. over i.p. administration. For instance, no significant protection was observed in the cortex, and striatal atrophy was only reduced by 25–30% after i.p. administration [14]. In contrast, i.n. administration of primary MSC-EVs in the present study resulted in a significant decrease of tissue atrophy by 50–60% in the striatum and up to 90% in the cortex. These results are supported by previous reports in rodent models of neonatal HI and in the combined setting of HI and systemic inflammation, both demonstrating significant protection by i.n. delivery of MSC-EVs [16, 40]. Although these findings suggest that the intranasal route is a less invasive and highly efficient therapy, anatomical and physiological differences of the nasal cavity between rodents and humans may hamper successful clinical translation of an intranasal MSC-EV therapy [70]. However, previous work in fetal sheep demonstrated protective effects of an i.n. MSC therapy on HI-induced white matter injury [41]. More recently, Robertson et al. showed a significant advantage of i.n. compared to i.v. delivery of MSCs in newborn piglets in the combined setting of HI and hypothermia, the obligatory clinical care for the treatment of neonatal HI-induced brain injury [39]. These encouraging proof-of-principle studies in large animal models support that it may be worth exploring i.n. delivery of stem cell-based therapies in neonates suffering from brain injury. Accordingly, a first clinical feasibility and safety trial in intranasally MSC-treated infants with neonatal stroke was recently successfully conducted [35].

The advantage of stem cell-based therapies over conventional pharmaceutical therapeutics is their multimodal mechanism of action [3, 4], probably explaining their high effectiveness in preclinical models of perinatal brain injury. Here, we show that the increased neuronal density in the striatum and cortex of ciMSC-EV-treated animals was closely associated with restauration of developmental proliferation in the subventricular zone (SVZ). The SVZ is one of the central regions of neurogenesis, juxtaposed to the striatum, where newly produced cells start their rostral and medial migration [71]. However, following an injury, they may also migrate towards lesioned areas, as suggested in an adult model of brain ischemia [72]. Therefore, increased neuronal numbers and structural tissue preservation in ciMSC-EV-treated HI-injured animals may result from prevention of both HI-induced neuronal cell death and disturbed neurogenesis in the SVZ. Neurogenesis-promoting effects of MSC-EV on neurogenesis have been described in different animal models, including stroke and spinal cord injury [8, 73], though the detailed mechanisms underlying these observations are still unclear. On the one hand, MSC-EVs may directly act on neural stem cells (NSC), as recently shown in vitro for placenta-derived MSC-EV products promoting NSC proliferation and activation [73]. On the other hand, the observation that MSC-EV-stimulated astrocytes release EVs that promote neurogenesis in an in vitro model of hypoxia–ischemia [74] points towards indirect effects. Though neurogenesis is a cardinal feature of SVZ-NSCs under physiological conditions, this differentiation program can be changed after an injury, redirecting SVZ-NSC differentiation towards glial cell lineages [75]. Further work will be needed to provide a direct link between improved proliferation in the SVZ and increased neuronal cell densities in the juxtaposed striatal and cortical regions following an i.n. ciMSC-EV treatment, e.g., by in vitro and in vivo cell fate tracing studies of NSCs. Interestingly, effects of ciMSC-EVs on HI-induced impaired proliferation in the second neurogenic niche of the CNS, the SGZ, were less pronounced, which may explain limited treatment efficiency on hippocampal tissue loss. Though speculative at this time point, one hypothesis for these regional differences might by a different EV distribution following intranasal treatment. The olfactory and trigeminal nerves, as well as the rostral migratory stream, were suggested as major nose-to-brain routes of i.n.-delivered EVs and drugs [76, 77]. The more rostral localization of the SVZ compared to the SGZ might result in a higher and faster accumulation of i.n.-delivered EVs in these brain regions. Furthermore, in contrast to the SGZ, the SVZ lines the lateral ventricles, with a subtype of NSCs (type B cells) having direct access to the CSF, which is supposed to be an alternative route of i.n.-delivered EVs [76]. High-resolution and kinetic EV-tracking analyses are required in future studies to delineate the immediate structural and cellular targets of i.n.-delivered ciMSC-EVs in perinatal brain injury.

Neuroprotective and neurorestorative effects of an ciMSC-EV application were accompanied by pronounced anti-inflammatory effects. The ability to modulate pro-inflammatory into regulatory immune responses is an important hallmark of potent MSC-EVs [78]. In accordance with previous work [14, 17, 40], we observed an overall reduced microglia activation following ciMSC-EV treatment, reflected by attenuation of HI-induced Iba-1 expression. Qualitative assessment of morphological changes showed that neonatal HI induces a hypertrophic and hyperramified phenotype, similarly as previously described in models of LPS-induced neuroinflammation and subarachnoid hemorrhage [54, 79]. These morphological alterations appeared to be less pronounced in ciMSC-EV-treated animals. Nevertheless, quantitative analyses would be needed to draw clear-cut conclusions on this feature of microglia activation. This requires other tissue preservations and most likely earlier time points of analyses considering massive microglia accumulation in severely injured animals observed at 7 days after injury in the present injury model. Furthermore, according to ongoing discussions about criteria to characterize the complex and divergent role of microglia in different pathophysiological conditions [80], multiple approaches including in vitro experiments should be followed. Thomi et al. showed that LPS-stimulated microglia co-cultured with MSC-EVs produce less pro-inflammatory cytokines [17]. In support of this, the present in vivo results show a decline in the expression of the pro-inflammatory cytokine IL-1-beta and an increased expression of the anti-inflammatory cytokines TGF-beta and IL-4 in brain tissue lysates of ciMSC-EV-treated animals. Taken together, our findings suggest that anti-inflammatory effects of ciMSC-EVs may have facilitated an environment limiting secondary injury processes and supporting regeneration [81].

In addition to local microglia, infiltrated leukocytes from the periphery contribute to the overall inflammatory micromilieu in the injured brain. In line with our observations in models of adult stroke [25, 34], we detected a reduced infiltration of peripheral immune cells following ciMSC-EV treatment. However, for neonatal as well as adult ischemic brain injury, it remains unclear whether this is caused by modulation of leukocytes in the periphery or by alterations of the blood brain barrier, e.g., through upregulation of adhesion molecules like VCAM-1, which are important for transmigration of peripheral leukocytes into injured tissues [10, 57, 58]. Our findings that ciMSC-EV treatment prevented HI-induced upregulation of the endothelial adhesion molecule VCAM-1 without affecting peripheral cytokine levels in HI-injured mice imply that reduced leukocyte infiltration following intranasal delivery of ciMSC-EVs was rather caused by inhibition of endothelial activation than by direct modulation of the peripheral immune system. This is in contrast to our previous report in adult stroke, where intravenous application of MSC-EVs modulated stroke-induced peripheral immune responses [8]. In addition to differences related to experimental models, age, and time points of analyses, different delivery routes may also play a role. In the former study, EVs were administered intravenously and can therefore directly modulate peripheral leukocyte responses. Furthermore, our in vitro analyses demonstrated a strong immunomodulatory activity of ciMSC-EVs when directly incubated with peripheral blood leukocytes. Therefore, the absence of obvious effects of i.n.-delivered ciMSC-EVs on the peripheral immune status in our in vivo experiments might be explained by the fact that they have less access to the peripheral immune system. In support of this, Thomi et al. showed that i.n.-administered MSC-EVs appear in the forebrain within the 0.5 and 3 h in the brain, while they were not detected in the spleen [16].

In addition to microglia and peripheral leukocytes, astrocytes contribute to neuroinflammatory responses in the brain. In the present work, we show that HI induces a strong increase of GFAP expression, a major hallmark of reactive astrocytes [63], which was significantly reduced after i.n. delivery of ciMSC-EVs. In response to an injury, a proportion of reactive astrocytes proliferate [63–65], which was confirmed in the present study and may partially explain increased GFAP abundance in HI-injured animals. While GFAP expression was reduced in ciMSC-EV-treated animals, astrocyte proliferation was not modulated. These results indicate that protective effects of ciMSC-EVs were rather related to modulation of the cells’ activity than to alteration of their proliferative response. However, the number of proliferating astrocytes per se was scarce at the selected time point of analysis, i.e., 7 days after injury. This is supported by previous work in neonatal rats showing that the astrocytic proliferative response at 7 days after HI was only half of that detected at 1 day after HI [82]. Additional analyses at earlier time points are needed to draw clear-cut conclusions about the impact of a i.n. ciMSC-EV treatment on HI-induced astrocyte proliferation. Furthermore, the concept of reactive astrocytes, purely based on proliferation, is increasingly challenged by several concepts characterizing astrocyte diversity in phenotype and function [63–65]. Though still debated, one of these concepts is the classification into pro-inflammatory A1 and anti-inflammatory A2 cells [53]. We observed a significant downregulation of the typical A1 marker C3 and an upregulation of the typical A2 marker S100A10, suggesting that ciMSC-EVs inhibit HI-induced alterations of astrocyte polarization towards a pro-inflammatory and neurotoxic A1 phenotype. This is supported by our mRNA expression analyses of important neurotrophic growth factors (i.e., BDNF, EGF, and VEGF), major effector molecules of astrocytes, which have been suggested to provide support for vascular and oligodendrocyte development but also regeneration in response to injury [83–86]. In line with that, we observed that i.n. delivery of ciMSC-EVs protects from HI-induced impairment of endothelial proliferation and oligodendrocyte maturation. Effects on the vasculature and endothelial proliferation appear highly relevant for neonatal brain injury, since physiological brain development and maintenance are highly dependent on proper vascularization not only for oxygen and nutrient supply but also for creating the scaffold necessary for neuronal and oligodendroglial migration and differentiation [87–89].

Our findings are of particular importance for clinical translation since they suggest a multimodal protective therapy with intranasal delivery of ciMSC-EVs for the treatment of neonatal encephalopathy caused by HI. The only available and obligatory therapy for these neonates is hypothermia, which is, however, limited due to a short therapeutic window of 6 h and limited efficacy in severe cases, so that 30% of cooled infants still suffer from major neurological problems [18, 19, 90]. Furthermore, HT has been shown to be ineffective in low- and middle-income countries [91]. With the present findings, we suggest a low invasive but highly effective therapy with a larger therapeutic window compared to HT. The standardized large-scale production of neuroprotective ciMSC-EVs offers novel opportunities for the treatment of HI-induced brain injury, not only in the combination with HT but also as an alternative stand-alone therapy in cases when HT is not applicable.

## Conclusion

In the present proof-of-principle study, we demonstrate that our immortalization procedure of MSCs apparently has no negative impact on the therapeutic properties of EVs. Importantly, ciMSC-EVs retain their multimodal neuroprotective capacities by suppression of neuro-inflammatory responses and prevention of disturbed endogenous repair and neurodevelopmental processes in neonatal HI-induced brain injury. Although the current findings present an important milestone in translating ciMSC-EV products into clinics, the robustness of the ciMSC-EV production process needs to be confirmed. This includes the investigation of batch-to-batch variations of ciMSC-EV preparations regarding their metric features and functionality in vitro and in vivo. Furthermore, despite the fact that we have identified an optimal delivery mode via the intranasal route, a central question to be answered in future studies is whether this therapy can be combined with, and overcomes current limitations of hypothermia, the standard clinical care for neonates with HI-induced brain injury.

## Supplementary Information

**Additional file 1: Supplementary Fig. 1.** ciMSCs retain bona fide MSC characteristics. **Supplementary Fig. 2.** MSC41.5-EV and ciMSC41.5 EV preparations contain CD9+ , CD63+ and CD81+ EVs. **Supplementary Fig. 3.** MSC41.5-EVs and ciMSC41.5-EVs comparably suppress activation of CD4 and CD8 T cells. **Supplementary Fig. 4.** Intranasal ciMSC-EV application does not modulate neonatal H-Iinduced proliferative responses in the cortex, striatum and subgranular zone of the hippocampus. **Supplementary Fig. 5.** Example image of Iba-1 staining in a severely affected mouse 7 days after HI. **Supplementary Fig. 6.** Original full-length western blot images used for representative illustrations.  **Supplementary Table 1.** Antibodies used in classical and imaging flow cytometry. **Supplementary Table 2.** Particle and protein characteristics of the applied EV preparations. **Supplemental Table 3.** Laser settings applied in imaging flow cytometry analyses. **Supplemental Table 4**. Compensation matrix applied in imaging flow cytometry analyses. **Supplementary Table 5.** Antibodies used for immunohistochemistry. **Supplementary Table 6.** TaqMan Assays used for mRNA expression analyses. **Supplementary Table 7.** Antibodies used for western blot analyses.

## Data Availability

All data generated or analyzed during this study are included in this published article and its supplementary information files.

## Abbreviations

aGvHD: Acute graft-versus-host disease
Arg-1: Arginase-1
BDNF: Brain-derived neurotrophic factor
C3: Complement C3
CC1: Adenomatous polyposis coli, clone CC1 positive
ciMSC-EV: EV from clonally expanded immortalized MSCs
CM: Conditioned medium
CNPase: 2′,3′-Cyclic-nucleotide 3′-phosphodiesterase
Cox-2: Cyclooxygenase 2
Dapi: 4′,6-Diamidin-2-phenylindol
EGF: Epidermal growth factor
EV: Extracellular vesicles
GAPDH: Glutaraldehyde-3-phosphate dehydrogenase
GFAP: Glial fibrillary acidic protein
HI: Hypoxia–ischemia
hPL: Human platelet lysate
hTERT: Human telomerase reverse transcriptase
i.n.: Intranasal
i.p.: Intraperitoneal
i.v.: Intravenous
Iba-1: Ionized calcium-binding adaptor protein-1
iNos: Inducible nitric oxide synthase
MBP: Myelin basic protein
mdMLR: Multi donor mixed lymphocyte reaction assay
MMP9: Matrix metalloproteinase 9
MSC: Mesenchymal stromal cells
NaCl: Sodium chloride
PBMC: Peripheral blood mononuclear cells
PBS: Phosphate-buffered saline
PFA: Paraformaldehyde
PTX3: Pentraxin 3
SDS: Sodium dodecyl sulfate
SGZ: Subgranular zone
SVZ: Subventricular zone
TGF-beta: Transforming growth factor-beta
TNF-alpha: Tumor necrosis factor-alpha
VCAM-1: Vascular cell adhesion molecule-1
VEGF: Vascular endothelial growth factor
YM-1: Chitinase 3-like 3 protein
