# Supplementary material for: Extracellular vesicles from immortalized mesenchymal stromal cells protect against neonatal hypoxic-ischemic brain injury
Source: Inflamm Regen. 2023 Apr 17;43:24. doi: 10.1186/s41232-023-00274-6 (PMC10108458; doi:10.1186/s41232-023-00274-6)
Supplement: Supplementary file 1 — Additional file 1: Supplementary Fig. 1. ciMSCs retain bona fide MSC characteristics. Supplementary Fig. 2. MSC41.5-EV and ciMSC41.5 EV preparations contain CD9+ , CD63+ and CD81+ EVs. Supplementary Fig. 3. MSC41.5-EVs and ciMSC41.5-EVs comparably suppress activation of CD4 and CD8 T cells. Supplementary Fig. 4. Intranasal ciMSC-EV application does not modulate neonatal H-Iinduced proliferative responses in the cortex, striatum and subgranular zone of the hippocampus. Supplementary Fig. 5. Example image of Iba-1 staining in a severely affected mouse 7 days after HI. Supplementary Fig. 6. Original full-length western blot images used for representative illustrations. Supplementary Table 1. Antibodies used in classical and imaging flow cytometry. Supplementary Table 2. Particle and protein characteristics of the applied EV preparations. Supplemental Table 3. Laser settings applied in imaging flow cytometry analyses. Supplemental Table 4. Compensation matrix applied in imaging flow cytometry analyses. Supplementary Table 5. Antibodies used for immunohistochemistry. Supplementary Table 6. TaqMan Assays used for mRNA expression analyses. Supplementary Table 7. Antibodies used for western blot analyses. [file 41232_2023_274_MOESM1_ESM.pdf]

Supplementary Material

Extracellular vesicles from immortalized mesenchymal stromal cells protect against neonatal hypoxic-ischemic brain injury

Nicole Labusek[1], Yanis Mouloud[2], Christian Köster[1], Eva Diesterbeck[1], Tobias Tertel[2], Constanze Wiek[3], Helmut Hanenberg[3,4], Peter A. Horn[2], Ursula Felderhoff-Müser[1], Ivo Bendix[1], Bernd Giebel[2#], Josephine Herz[1#]

[1]Department of Pediatrics I, Neonatology & Experimental perinatal Neurosciences, Centre for Translational and Behavioral Sciences (C-TNBS), University Hospital Essen, University Duisburg-Essen, Essen, Germany

[2]Institute for Transfusion Medicine, University Hospital Essen, University of Duisburg-Essen, Essen, Germany

[3]Department of Otorhinolaryngology and Head/Neck Surgery, University Hospital Düsseldorf, Heinrich-Heine-University, Düsseldorf, Germany

[4]Department of Pediatrics III, University Hospital Essen, University of Duisburg-Essen, Essen, Germany

[#] correspondence to: Bernd Giebel and Josephine Herz

Supplementary Figures: 6

Supplementary Tables: 7

Supplementary Figures

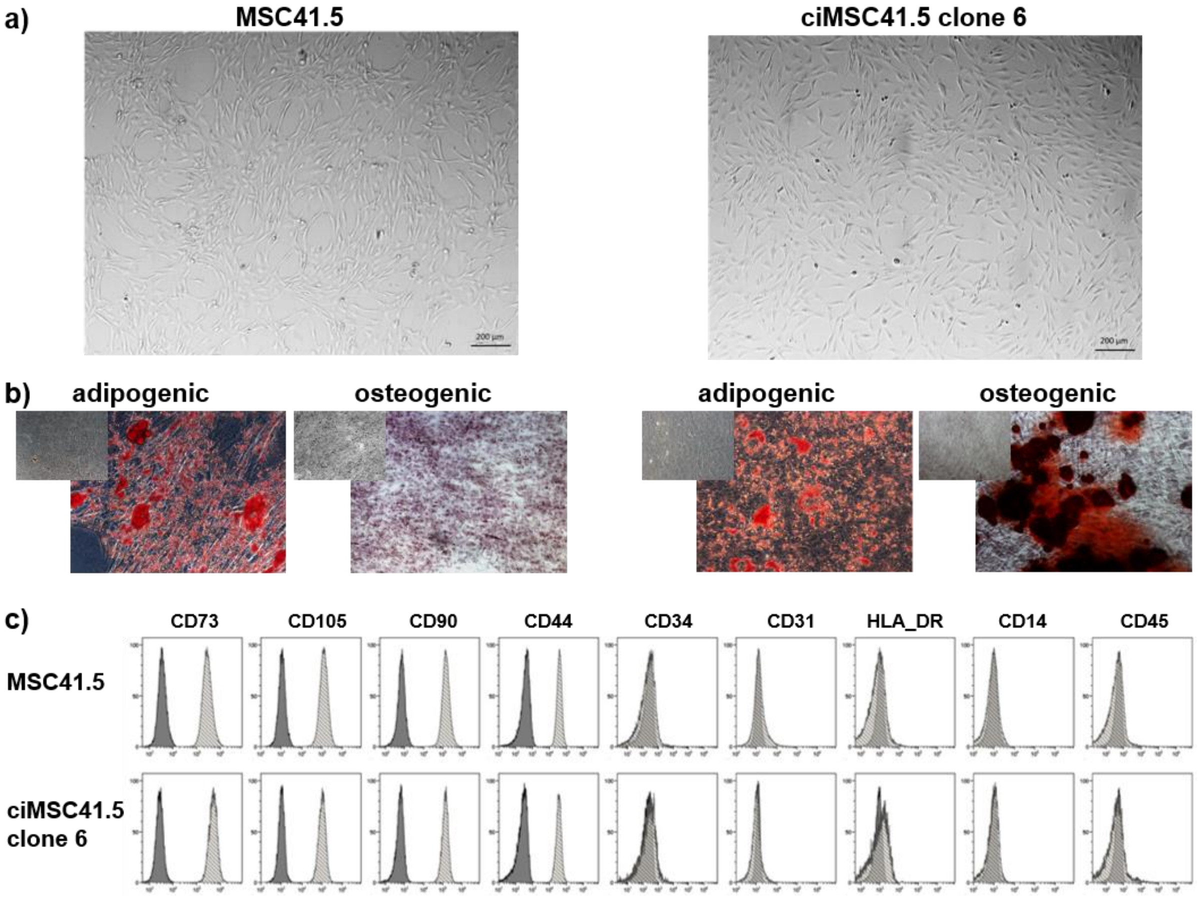

Supplementary Figure 1: ciMSCs retain *bona fide* MSC characteristics. Morphology of MSC41.5 cells (left) were compared to their clonally expanded, immortalised derivatives ciMSC41.5 clone 6 (right) as they appear in multilayered cell factories (a). Osteogenic and adipogenic differentiation capabilities of primary MSCs and ciMSCs were documented (b); inlets show images of non-induced but stained MSCs. Cell surface antigen expression of *bona fide* MSC antigens and negative markers was analysed via flow cytometry to compare the phenotype of primary and ciMSCs (c).

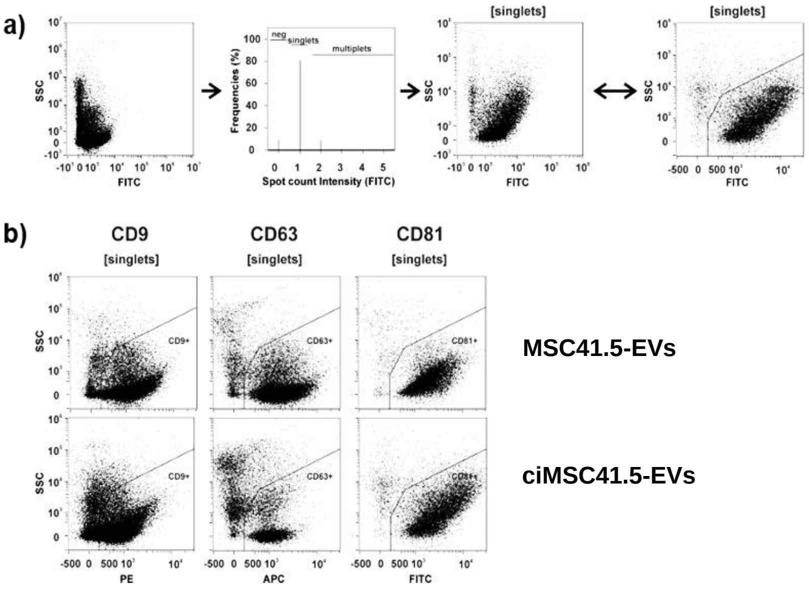

Supplementary Figure 2: MSC41.5-EV and ciMSC41.5 EV preparations contain CD9^{+}, CD63^{+} and CD81^{+} EVs. EV preparations were analyzed by imaging flow cytometry. All recorded signals (scatter to fluorescence signal, left plot in a) were discriminated according to the number of objects that could be identified. Only signals containing exactly one object (singlets) were identified and are depicted in zoomed images of the plots (a, [singlets]). Side scatter and fluorescence intensity plots of single objects labelled with either anti-CD9, anti-CD63 or anti-CD81 are shown for MSC41.5-EV and ciMSC41.5-EV preparations (b).

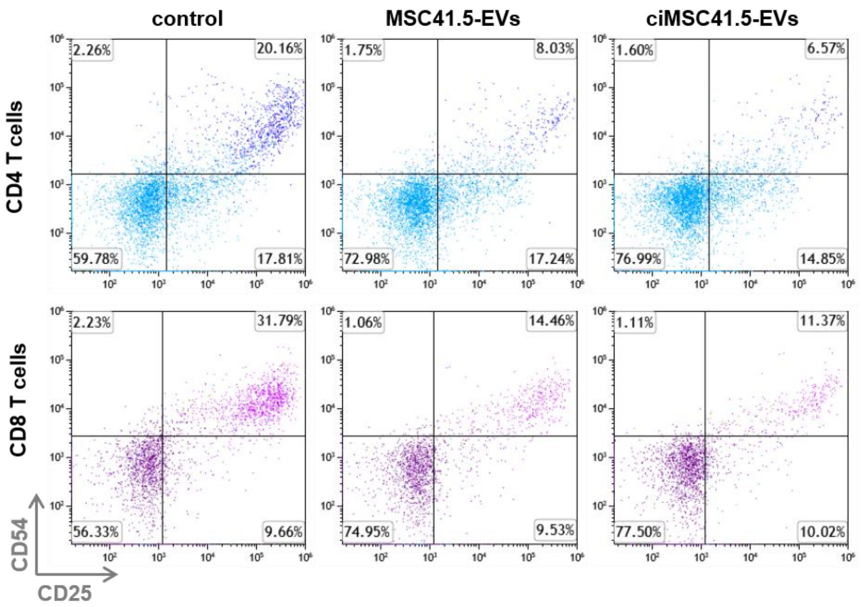

Supplementary Figure 3: MSC41.5-EVs and ciMSC-41.5-EVs comparably suppress activation of CD4 and CD8 T cells. The immunomodulatory potential of EVs derived from primary MSC41.5 and ciMSC41.5 to suppress allogeneic activation of human CD4 and CD8 cells was evaluated in a multi-donor mixed lymphocyte reaction assay (Nardi Bauer et al., 2023). Here 600.000 cells of a mononuclear cell mixture derived from peripheral blood samples of 12 healthy donors were cultured for 5 days, either in the presence or absence of 25 µg of given EV preparations. Cells were labelled with anti-CD4, anti-CD25 and anti-CD54 antibodies. T cells were gated as $CD4^+$ or $CD8^+$ living cells and activated T cells were identified as $CD25^+CD54^+$ cells by flow cytometry.

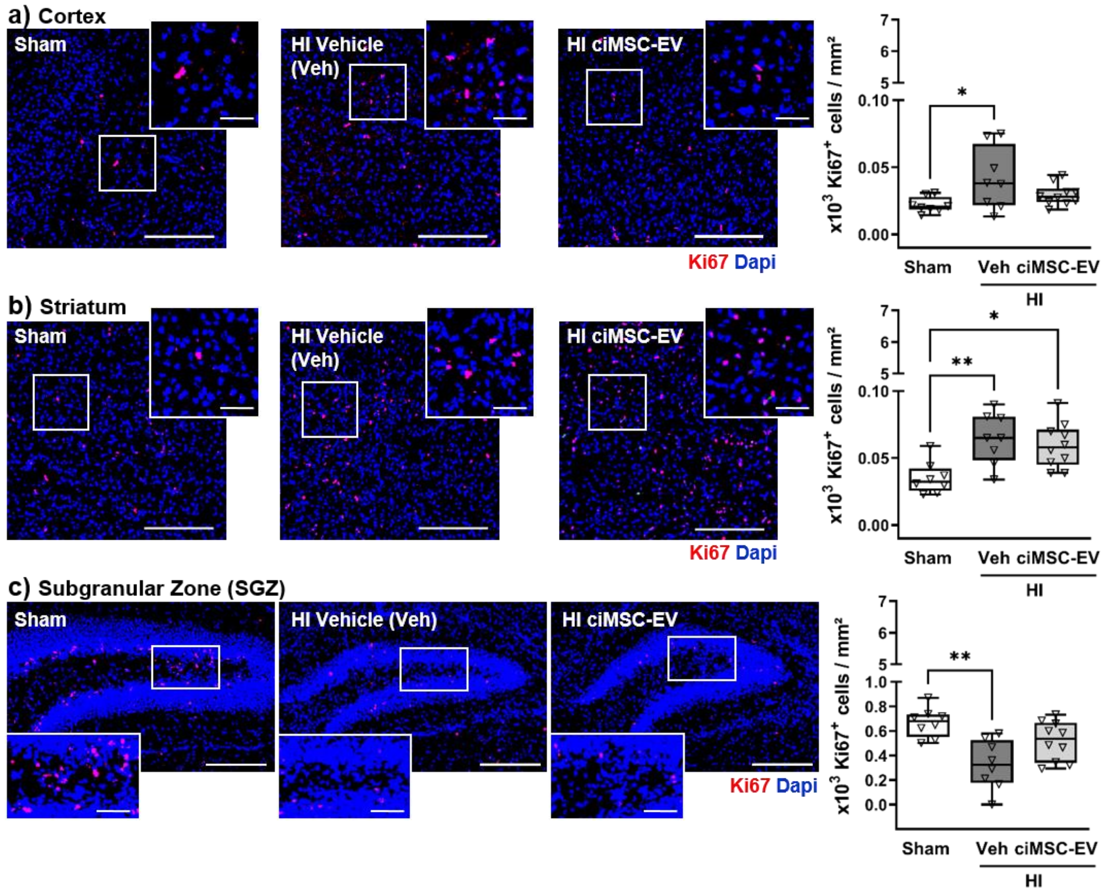

Supplementary Figure 4: Intranasal ciMSC-EV application does not modulate neonatal HI-induced proliferative responses in the cortex, striatum and subgranular zone of the hippocampus. Cellular proliferation was analyzed via immunohistochemistry for Ki67 in the cortex (a), striatum (b) and subgranular zone (c) in tissue sections from 16-day-old C57BL/6 mice that were exposed to HI on postnatal day 9 followed by i.n. delivery of 0.9% NaCl (Vehicle, Veh) or ciMSC-EVs 1, 3 and 5 days after HI. In large scale representative images (scale bar: 200 µm), insets show higher magnification images (scale bar: 50 µm) of rectangles depicted in low magnification images. *$p < 0.05$, **$p < 0.01$, n = 8-10/group.

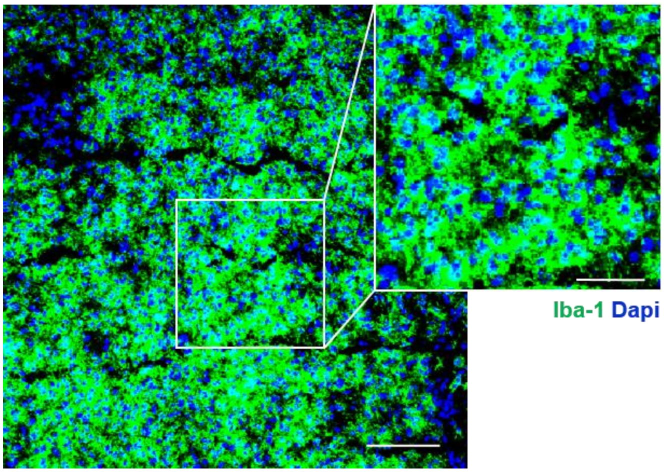

Supplementary Figure 5: Example image of Iba-1 staining in a severely affected mouse 7 days after HI. Microglia activation was analyzed via immunohistochemistry for Iba-1 in native fresh frozen tissue sections 7 days after neonatal HI. The representative image is derived from a vehicle-treated severely affected HI-injured animal to demonstrate dense accumulation of microglia, not allowing identification of single Iba-1 positive cells or quantification of morphological changes. The inset shows a higher magnification image (scale bar: 50 µm) of the rectangle depicted in the low magnification image (scale bar: 100 µm).

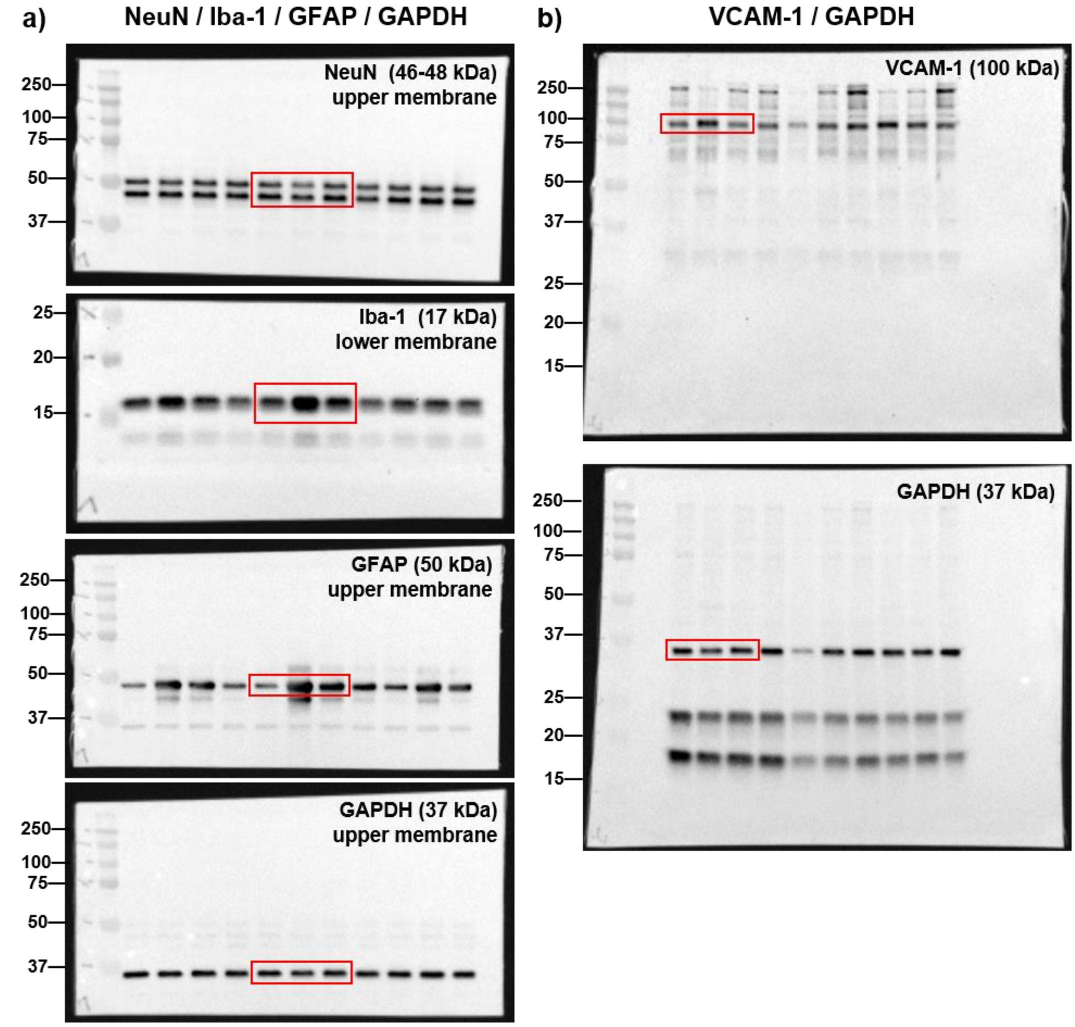

Supplementary Figure 6: Original full-length western blot images used for representative illustrations. All images represent merged images of original western blot images and transmitted light images to visualize ladder bands. Analysis of NeuN, Iba-1 and GFAP was performed on the same membrane without stripping (a). To avoid cross reactions with secondary antibody binding of NeuN and Iba-1 (both rabbit host, Suppl. Table 7), membranes were cut at approximately 30 kDa prior to antibody incubation and detection (a). For detection of VCAM-1 a separate western blot was performed (b). Red boxes indicate cropped regions used for illustrations in figures of the main manuscript.

Supplementary Tables

Supplementary Table 1: Antibodies used in classical and imaging flow cytometry

Antigen	Dilution	Conjugat	Host	Isotyp	Clone	Supplier	Catalog no.
Human CD14	1:50	PO	Mouse	IgG_1	MEM-15	Exbio	PO-293-T100
Human CD31	1:50	PE	Mouse	IgG_1	1F11	Beckman Coulter	IM2409
Human HLA-DR	1:50	ECD	Mouse	IgG_1	Immu-357	Beckman Coulter	B92438
Human CD34	1:50	APC 750	Mouse	IgG_1	581	Beckman Coulter	B92463
Human CD44	1:50	APC	Mouse	IgG_{2bk}	G44-26	BD Biosciences	559942
Human CD45	1:50	BV 785	Mouse	IgG_{1k}	HI30	BioLegend	304048
Human CD73	1:50	FITC	Mouse	IgG_{1k}	AD2	BD Biosciences	561254
Human CD90	1:50	BV 605	Mouse	IgG_{1k}	5E10	BioLegend	328128
Human CD105	1:50	BV 421	Mouse	IgG_{1k}	43A3	BioLegend	323219
Human CD25	1:30	PE	Mouse	IgG_{1k}	BC-96	Thermo Fisher Scientific	12-0259- 42
Human CD54	1:30	AF 700	Mouse	IgG_{2b}	1H4	Exbio	A7-429-T100
Human CD4	1:30	BV 785	Mouse	IgG_{1k}	RPA-T4	Biolegend	300554
Human CD8	1:30	BV 650	Mouse	IgG_{1k}	SK1	Biolegend	344730
Human CD9	1:10	PE	Mouse	IgG_1	MEM-61	Exbio	1P-208-T100
Human CD63	1:10	APC	Mouse	IgG_1	MEM-259	Exbio	1A-343-T100
Human CD81	1:10	FITC	Mouse	IgG_{2a}	JS-64	Beckman Coulter	B25329

APC: Allophycocyanin, FITC: Fluorescein isothiocyanate, BV: Brilliant Violet, PO: Pacific Orange; PE = Phycoerythrin

Supplementary Table 2: Particle and protein characteristics of the applied EV preparations

	MSC41.5-EVs	ciMSC41.5-EVs
Particle number / ml	2.5×10^{11}	1.9×10^{11}
Particle diameter (mm)	116.3	109.6
Protein concentration (mg/ml)	5.5	4.7
Purity (particle number / mg protein)	4.5×10^{10}	4.04×10^{10}

Supplemental Table 3: Laser settings applied in imaging flow cytometry analyses

Laser [nm]	Used Power [mW]	Max. Power [mW]	Filter [nm]
375	70	70	-
488	100	100	FITC (Ch02) 480-560
561	200	200	PE (Ch03) 560-595
648	150	150	APC (Ch11) 642-745
785 (SSC)	70	70	SSC (Ch06) 756-780

Supplemental Table 4: Compensation matrix applied in imaging flow cytometry analyses

	Ch1	Ch2	Ch3	Ch4	Ch5	Ch6	Ch7	Ch8	Ch9	Ch10	Ch11	Ch12
**Ch1**	1	0.029	0.042	0	0	0	0	0	0	0	0.002	0
**Ch2**	0.051	1	0.05	0	0	0	0	0	0	0	0.002	0
**Ch3**	0	0.13	1	0	0	0	0	0	0.02	0	0.002	0
**Ch4**	0	0.064	0.49	1	0	0	0	0	0	0	0.003	0
**Ch5**	0	0.017	0.155	0	1	0	0	0	0	0	0.074	0
**Ch6**	0.015	0.02	0.04	0	0	1	0	0	0	0	0.01	0
**Ch7**	0.023	0.003	0.003	0	0	0	1	0	0.015	0	0.024	0
**Ch8**	0	0.032	0.008	0	0	0	0	1	0.012	0	0.023	0
**Ch9**	0	0.004	0.084	0	0	0	0	0	1	0	0.024	0
**Ch10**	0	0.002	0.041	0	0	0	0	0	0.084	1	0.028	0
**Ch11**	0	0.001	0.012	0	0	0	0	0	0.025	0	1	0
**Ch12**	0	0	0.003	0	0	0	0	0	0.013	0	0.125	1

Supplementary Table 5: Antibodies used for immunohistochemistry

Antigen	Dilution	Reactivity	Host	Supplier	Catalog no.
NeuN	1:500	mouse	Rabbit	Millipore	ABN78
CD31	1:100	mouse	Rat	BD Biosciences	550274
Olig2	1:100	mouse	Rabbit	Millipore	AB9610
Ki67	1:250	mouse/rat	Rabbit	Abcam	ab66155
Ki67*	1:100	mouse	Rat	Thermo Scientific	14-5698-82
APC-CC1	1:100	mouse/rat	Mouse	Calbiochem	OP80
Iba-1	1:500	mouse/rat	Rabbit	Wako	019-19741
GFAP	1:500	mouse	Mouse	Convance	SMI-22
CD45	1:100	mouse	Rat	BD Pharmingen	550539
C3	1:50	Mouse	Rat	Abcam	ab11862

**for co-labelling with Olig2*

Supplementary Table 6: TaqMan Assays used for mRNA expression analyses

Gene	Assay ID
arg-1	Mm00475988_m1
b-2m	Mm00437762
bdnf	Mm01334043_m1
c3	Mm01232779_m1
cc1	Mm00545877_m1
cnpase	Mm01306640_m1
cox-2	Mm03294838_g1
egf	Mm00438696_m1
il-1 beta	Mm00434228_m1
il-18	Mm00434226_m1
iL-4	Mm00445259_m1
inos	Mm00440502_m1
mbp	Mm01266402_m1
ptx-3	Mm00477268_m1
s100a10	Mm00501458_g1
serping 1	Mm00437835_m1
tgf-beta	Mm01178820_m1
vegf	Mm00437306_m1
ym-1	Mm00657889_mH

Supplementary Table 7: Antibodies used for western blot

Antigen	Dilution	Reactivity	Host	Supplier	Catalog no.
VCAM	1:1000	mouse	goat	R&D	AF643
Iba1	1:1000	mouse/rat	rabbit	WAKO	016-20001
NeuN	1:2000	mouse/rat	rabbit	Millipore	ABN78
GFAP	1:5000	mouse	mouse IgG2b	Convance	SMI 22
GAPDH	1:2000	mouse	Mouse IgM	Sigma	G8795

References

Nardi Bauer, F., Tertel, T., Stambouli, O., Wang, C., Dittrich, R., Staubach, S., Borger, V., Hermann, D.M., Brandau, S., and Giebel, B. (2023). CD73 activity of mesenchymal stromal cell-derived extracellular vesicle preparations is detergent-resistant and does not correlate with immunomodulatory capabilities. Cytotherapy 25, 138-147.
